# Quantitative genome-wide association study of six phenotypic subdomains identifies novel genome-wide significant variants in autism spectrum disorder

**DOI:** 10.1038/s41398-020-00906-2

**Published:** 2020-07-05

**Authors:** Afsheen Yousaf, Regina Waltes, Denise Haslinger, Sabine M. Klauck, Eftichia Duketis, Michael Sachse, Anette Voran, Monica Biscaldi, Martin Schulte-Rüther, Sven Cichon, Markus Nöthen, Jörg Ackermann, Ina Koch, Christine M. Freitag, Andreas G. Chiocchetti

**Affiliations:** 1 Department of Child and Adolescent Psychiatry, Psychosomatics and Psychotherapy, University Hospital Frankfurt, Goethe University, Frankfurt am Main, Germany; 2 Division of Molecular Genome Analysis and Division of Cancer Genome Research, German Cancer Research Center (DKFZ), Heidelberg, Germany; 3 Department of Child and Adolescent Psychiatry, Saarland University, Homburg, Germany; 4 Department of Child and Adolescent Psychiatry, University Hospital Freiburg, Freiburg, Germany; 5 Translational Brain Medicine, Department of Child and Adolescent Psychiatry, Psychosomatics, and Psychotherapy, RWTH Aachen University, Aachen, Germany; 6 JARA-BRAIN, Aachen, Germany; 7 Institute of Neuroscience and Medicine (INM-1), Research Center Juelich, Juelich, Germany; 8 Human Genomics Research Group and Division of Medical Genetics, Department of Biomedicine, University of Basel, Basel, Switzerland; 9 Institute of Medical Genetics and Pathology, University Hospital Basel, Basel, Switzerland; 10 Department of Genomics, University of Bonn, Bonn, Germany; 11 Molecular Bioinformatics, Institute of Computer Science, Johann Wolfgang Goethe-University Frankfurt am Main, Frankfurt am Main, Germany

**Keywords:** Genetics, Psychology

## Abstract

Autism spectrum disorders (ASD) are highly heritable and are characterized by deficits in social communication and restricted and repetitive behaviors. Twin studies on phenotypic subdomains suggest a differing underlying genetic etiology. Studying genetic variation explaining phenotypic variance will help to identify specific underlying pathomechanisms. We investigated the effect of common variation on ASD subdomains in two cohorts including >2500 individuals. Based on the Autism Diagnostic Interview-Revised (ADI-R), we identified and confirmed six subdomains with a SNP-based genetic heritability *h*^2^_*SNP*_ = 0.2–0.4. The subdomains nonverbal communication (NVC), social interaction (SI), and peer interaction (PI) shared genetic risk factors, while the subdomains of repetitive sensory-motor behavior (RB) and restricted interests (RI) were genetically independent of each other. The polygenic risk score (PRS) for ASD as categorical diagnosis explained 2.3–3.3% of the variance of SI, joint attention (JA), and PI, 4.5% for RI, 1.2% of RB, but only 0.7% of NVC. We report eight genome-wide significant hits—partially replicating previous findings—and 292 known and novel candidate genes. The underlying biological mechanisms were related to neuronal transmission and development. At the SNP and gene level, all subdomains showed overlap, with the exception of RB. However, no overlap was observed at the functional level. In summary, the ADI-R algorithm-derived subdomains related to social communication show a shared genetic etiology in contrast to restricted and repetitive behaviors. The ASD-specific PRS overlapped only partially, suggesting an additional role of specific common variation in shaping the phenotypic expression of ASD subdomains.

## Introduction

Autism spectrum disorder (ASD) is a phenotypically heterogeneous neurodevelopmental disorder. The diagnostic criteria according to DSM-5 (Diagnostic and Statistical Manual-5)^1^ include two symptom domains: (A) social communication and interaction and (B) restricted or repetitive patterns of behavior and interests. The genetic architecture of ASD is highly complex comprising common, rare inherited and de novo genetic variants. Common variants show small effects, but collectively have a substantial impact on ASD susceptibility explaining ~50% of ASD liability^2^. Phenotypic subdomains with high heritability^3^ and rather low cross-trait genetic correlation estimates are reported previously^4^. Albeit high heritability estimates in ASD, the genetic and biological contribution of individual ASD domains remains largely unknown. This can be attributed to its heterogeneous genetic and phenotypic complex architecture. An approach to address this difficulty and ravel the ASD complexity is to focus on ASD phenotypic domains and subdomains, which have been proposed to reduce genetic heterogeneity and thus increase statistical power^5^.

The phenotypic independence of the two DSM-5 dimensions has been shown previously^6^. Categorizing these domains further into independent phenotypic subdomains has shown evidence for an underlying strong genetic susceptibility as published by Liu et al.^3^. Based on the diagnostic algorithm items of the Autism Diagnostic Interview-Revised (ADI-R), they identified 6 subdomains in the Autism Genome Project (AGP) cohort, namely joint attention (JA), social interaction and communication (SI), nonverbal communication (NVC), and peer interaction (PI) related to domain A, and repetitive sensory-motor behavior (RB), compulsion/restricted interests, or insistence on sameness (RI) related to domain B. Linkage-based common-variant heritability of these ASD subdomains ranged between 29% (PI) and 65% (RI), which is comparable to the additive SNP-based heritability of 40–60%^2^ or the twin-based additive genetic heritability of 62–81%^3,4^ of the categorical ASD diagnosis.

ASD domains and subdomains are likely to show distinct underlying genetic risk. Twin studies reported the genetic correlation (*r*_*g*_) between domain A and domain B to be ranging between ~10 and 50%, and varying between males and females^4^. Another study investigating 189 twins with at least one affected individual reported that the overall co-twin co-trait correlations were small between five phenotypic subdomains derived from the Development and Wellbeing Assessment instrument^7^ (i.e., social, communication, restricted repetitive behavior and interests, language development, and insistence on sameness (IS))^8^.

The high genetic heritability and the low genetic correlation between domains and subdomains suggest that the previously reported statistically independent ADI-R subdomains^3,9^ are also genetically independent. Evidence for a genetic etiology further comes from a genome-wide SNP-based linkage study on these subdomains^3^, which identified two genetic loci, i.e., for JA (11q23) and RB (19q13.3). In addition, numerous quantitative genome-wide association studies (qGWAS) have focused on different ASD-related dimensional traits derived from the ADI-R diagnostic algorithm (e.g., repetitive sensory-motor behavior or IS)^10^, the SRS total score^11^, or single items of the ADI-R^12^, the ADOS and the SRS^12^. None of the reported genome-wide significant findings were observed in another ASD subdomain or trait, or independently replicated in any study. This may be attributed to small genetic effects or limited sample size^13^; still, it may also indicate a differing underlying genetic etiology and implicated neurobiological mechanisms of different ASD subdomains.

In addition, an overlap with common genetic risk for ASD as categorical diagnosis has not been assessed in previous studies. To capture the additional value of studying phenotypic subdomains, their genetic correlation with ASD as a categorical diagnosis also needs to be explored. Regarding the small genetic effect of single SNPs, a powerful approach to capture the role of common genetic variation is to study the combined effect of SNPs by a polygenic risk score (PRS). This approach has been taken in ASD research to identify cross-disorder genetic risk, to study the role of common variation in different ASD subtypes, such as low and high IQ^14^, and genetic overlap with different traits observed in the population^15^. Given the polygenic etiology of ASD as categorical diagnosis and the assumed differential polygenic etiology of variance in phenotypic subdomains, it is of prime interest to study the overlap of general polygenic risk on ASD with specific genetic risk for the subdomains.

Regarding the implicated neurobiology, similarly to the assumed differential genetic risk, specific underlying neurobiological mechanisms are expected for the different ADI-R algorithm-based subdomains. Studies on neurobiological mechanisms in ASD as categorical diagnosis converge with regard to abnormal neuronal function and early-age brain growth abnormality^16^. ASD-associated genes are implicated in synaptic scaffolding, neuronal transmission, chromatin remodeling, protein synthesis or degradation, or actin cytoskeleton dynamics^14^. Previous research has also shown that ASD-associated genes are involved in numerous biological processes, such as the mammalian target of rapamycin (mTOR)^17^, Wnt^18^, and calcium (Ca2 + ) signaling pathways^19^. Although these pathways are well known for their role in ASD, there is still a great need to understand how dysregulation of these pathways is involved in modulating the subdomains of ASD. From a biological perspective, we hypothesize that the phenotypic domains of social interaction and stereotyped behavior show differential underlying pathomechanisms. This assumption is based on the observation that genetic animal models for ASD show inconsistent phenotypes. For example, Nlgn3 (Neuroligin) adult knockout (KO) mouse model showed normal direct social interaction, but was engaged in repetitive behavior^20^, whereas Nrxn2α (Neurexin 2α) KO mice showed social deficits, but did not exhibit stereotyped repetitive behavior^21^. Moreover, magnetic resonance imaging (MRI) studies in humans have shown that inferior frontal gyrus, amygdala, prefrontal, and temporal cortices are related to defects in social language processing and social attention^22,23^, whereas the orbitofrontal cortex and basal ganglia have been associated with repetitive and stereotyped behavior of ASD^24^. Given the concept of ASD as an early developmental disorder, another biologically plausible argument for a differential genetic regulation of ASD-related subdomains stems from the finding of distinct transcriptomic signatures during development of these brain regions.

We hypothesize that distinct common genetic variants will modulate ADI-R-derived ASD subdomains, which are related to specific underlying biological processes, and gene-regulatory signatures. Thus, we performed a qGWAS on ADI-R-derived ASD subdomains dissecting their genetic etiology, and investigated their relation to the polygenic risk for ASD.

## Materials and methods

### Study cohort

We included a German (DE) cohort (*n* = 625 trios, *n* = 53 duos, and *n* = 27 singletons) and the AGP cohort (*n* = 2730 trios and *n* = 5 duos). Diagnosis was based on thorough clinical assessment using Social Communication Questionnaire (SCQ), ADI-R, and/or ADOS. Exclusion criteria and QC were based on the AGP cohort^25^. For final analysis, only the index patients (AGP *n* = 1,895, DE *n* = 614) with ADI-R and genotype information available were included (Supplementary Material).

### Genotype data

DE-cohort samples were genotyped on Illumina Human Omni Express 12v1-H chips. AGP samples were categorized into stage 1 and 2 samples, genotyped on 550 K Illumina, 510 K Illumina, 1 M Single, and 1 M Duo Illumina chips. However, all the stage 1 and 2 samples included in this study were genotyped on 1 M Illumina chips. We performed quality checks of both datasets separately. Genotype imputation was based on *minimac3*^26^. For detailed procedure and power analysis see Supplementary Material and Yousaf et al.^27^.

### Statistical analysis

All statistical analyses were performed in R-3.4.4 if not otherwise specified. For an overview of the analyses and the cohorts used for those analyses, refer to Supplementary Fig. 1.

#### Imputation of phenotype data

From Liu et al.^3^, we selected the 28 “ever/most abnormal” items from the ADI-R questionnaire available for verbal and nonverbal individuals. Individuals with >10% missing items were excluded. Missing scores were imputed using multivariate imputation by chained equations (MICE) applying predictive mean matching (pmm) in R package *mice*^28^ (Supplementary Material).

#### Define ADI-R subdomains

Subdomains were identified based on ADI-R data of the AGP cohort using principal component analysis with “varimax” rotation in R package *psych*^29^ as published^10^. Components were selected based on the Kaiser criterion. Confirmatory factor analysis (CFA) was performed in the DE cohort implementing R package *lavaan*^30^. For identified components, the sum of items with loading above 0.4 was calculated (Supplementary Material).

### Single-nucleotide polymorphisms (SNP)-based analysis

The implemented algorithms require large sample sizes. To increase power, SNP-based analyses were performed in the combined cohort (AGP and DE). For quantitative GWAS, the sample size had a power of 1-beta > 80% to explain 6% of the variance (*R*² = 0.06) in the DE cohort, 1.5% in the AGP cohort, and 1.2% in the combined cohort with a genome-wide significance threshold of alpha = 5e^−8^. Power analysis was performed using Quanto (http://biostats.usc.edu/Quanto.html). See the power analysis for performing genetic heritability in the supplementary material.

#### Polygenic risk scores (PRS)

To identify the shared etiology between an ASD diagnosis and the phenotypic subdomains, we performed a polygenic risk score analysis implementing the Psychiatric Genomics Consortium (PGC) summary statistics of ASDs (see http://pgc.unc.edu). Polygenic risk score analysis was performed using PRSice tool^31^ (Supplementary Material) in the merged cohort. *P* values for shared etiology were corrected using false discovery rate (FDR).

#### Genetic heritability and its correlation

SNP-based heritability (*h*^2^_*SNP*_) was calculated using the GCTA software^32^ based on the genetic relationship matrix (GRM) between pairs of individuals. For genetic correlation (*r*_*g*_) analysis, bivariate genomic GREML analysis was performed in GCTA (Supplementary Material) using the merged cohort.

#### Quantitative GWAS

SNP-based association analysis was performed in combined as well as individual cohorts. However, for further downstream analyses, we only used findings replicated in the GWAS of individual cohorts. Linear mixed-effect regression models with the subdomains as dependent variables were applied with fixed effects for gender, age, first four dimensions of the multidimensional scaling results (population stratification) from plinkv1.9^33^ (Supplementary Fig. 2), and with recruitment site of individuals as a random effect. The analysis was implemented using R package *lme4*^34^. Due to the high amount of missing IQ values, we did not correct for IQ. Correlations between IQ and the subdomains were minimal in both samples (cor = −0.26–0.12).

### Gene-based analysis

#### Gene-based association

This analysis was performed separately on the individual cohorts based on their respective GWAS output. The simultaneous joint effect of multiple SNPs was determined using Multimarker Analysis of GenoMic Annotation (MAGMA) software package *v1.06*^35^. qGWAS results of the individual cohorts were used. To reduce false-positive findings, we included only genes with *P*_permuted_ ≤ 0.05 replicated in both datasets for further analysis.

#### Pathway and brain network analysis

The significant (*P*_permuted_ <0.05) and overlapping genes from the MAGMA analysis resulting from both the cohorts were subjected to these analysis. Gene ontology (GO) and pathway analysis was performed using GO-Elite^36^. Brain network analysis was based on published gene lists of the 29 transcriptome modules (kindly provided by Dr. Kang) co-regulated during the development of the human brain^37^. Replicated genes from MAGMA analysis for each subdomain were tested for enrichment using Fisher-exact test.

## Results

In our study, we refer domain A as the ASD domain “Social interaction and social communication” domain, whereas domain B refers to the “restricted repetitive behaviors, interests, and activities” in ASD. The quantitative traits in our study can be classified into either domain A or domain B, i.e., SI, JA, PI, and NVC belong to domain A, whereas RB and RI belong to domain B.

### Descriptive data

Complete phenotypes and genotypes (*N* = 6,900,500 SNPs) were available for 1895 AGP and 614 DE cases with no difference in gender distribution across cohorts (*P* = 0.373). The DE cohort was older at diagnosis and showed a higher IQ compared with AGP sample (Table 1).

**Table 1:** Descriptive statistics of samples with complete phenotype and genotype data.

	AGP	DE	*P*	Merged
*N* total	1895	614		2509
Age at diag. in months, mean (SD)	103.11 (58.52)	128.72 (74.19)	<0.001^a^	109.38 (63.66)
Male gender, *N* (%)	1649 (87.02%)	525 (85.50%)	0.373^b^	2174 (86.64%)
Female gender, *N* (%)	246 (12.98%)	89 (14.50%)		335 (13.35%)
IQ, mean (SD)	78.63 (24.44)	88.96 (23.30)	<0.001^a^	80.99 (24.56)
IQ > 70, *N* (%)	1145 (60.42%)	418 (74.51%)	<0.001^b^	1563 (62.29%)
IQ ≤ 70, *N* (%)	750 (39.58%)	143 (25.50%)		893 (35.59%)
*Subdomains, mean (SD)*				
Social interaction (SI)	10.17 (3.24)	10.30 (3.43)	0.180^a^	10.20 (3.29)
Joint attention (JA)	12.86 (4.63)	11.92 (5.02)	<0.001^a^	12.63 (4.74)
Peer interaction (PI)	7.31 (2.56)	7.30 (2.79)	0.815^a^	7.31 (2.61)
Nonverbal communication (NVC)	4.14 (2.24)	4.37 (2.21)	0.023^a^	4.19 (2.23)
Repetitive sensory-motor behavior (RB)	6.04 (2.97)	5.16 (3.25)	<0.001^a^	5.83 (3.07)
Restricted interests (RI)	3.08 (2.03)	2.91 (1.90)	0.103^a^	3.04 (2.00)

*DE* German cohort, *AGP* Autism Genome Project cohort, *diag.* diagnosis, *SD* standard deviation.

^a^Wilcoxon test.

^b^Chi-square test.

P: nominal *P* value comparing DE versus AGP cohort.

### ADI-R algorithm-based subdomains

The AGP cohort satisfied the sample adequacy criteria (Supplementary Table 1). Six subdomains (Supplementary Table 2, Supplementary Fig. 3) were identified and labeled as SI (five items), JA (eight items), PI (four items), NVC (three items), RB (five items), and RI (three items). The item “*Conventional/Instrumental gestures*” loaded on SI and NVC, respectively, and—in accordance with the previously published study^3^—was included into NVC. CFA in the DE cohort confirmed the structure (Supplementary Table 3). No differences with respect to SI, PI, and RI were observed between cohorts (*P*_all_ > 0.1). JA and RB were lower in the DE compared with the AGP cohort, while NVC was higher (*P*_all_ < 1 × 10^−03^, Table 1).

### Single-nucleotide polymorphism (SNP)-based analysis

#### Polygenic risk scores (PRS)

**Fig. 1:**
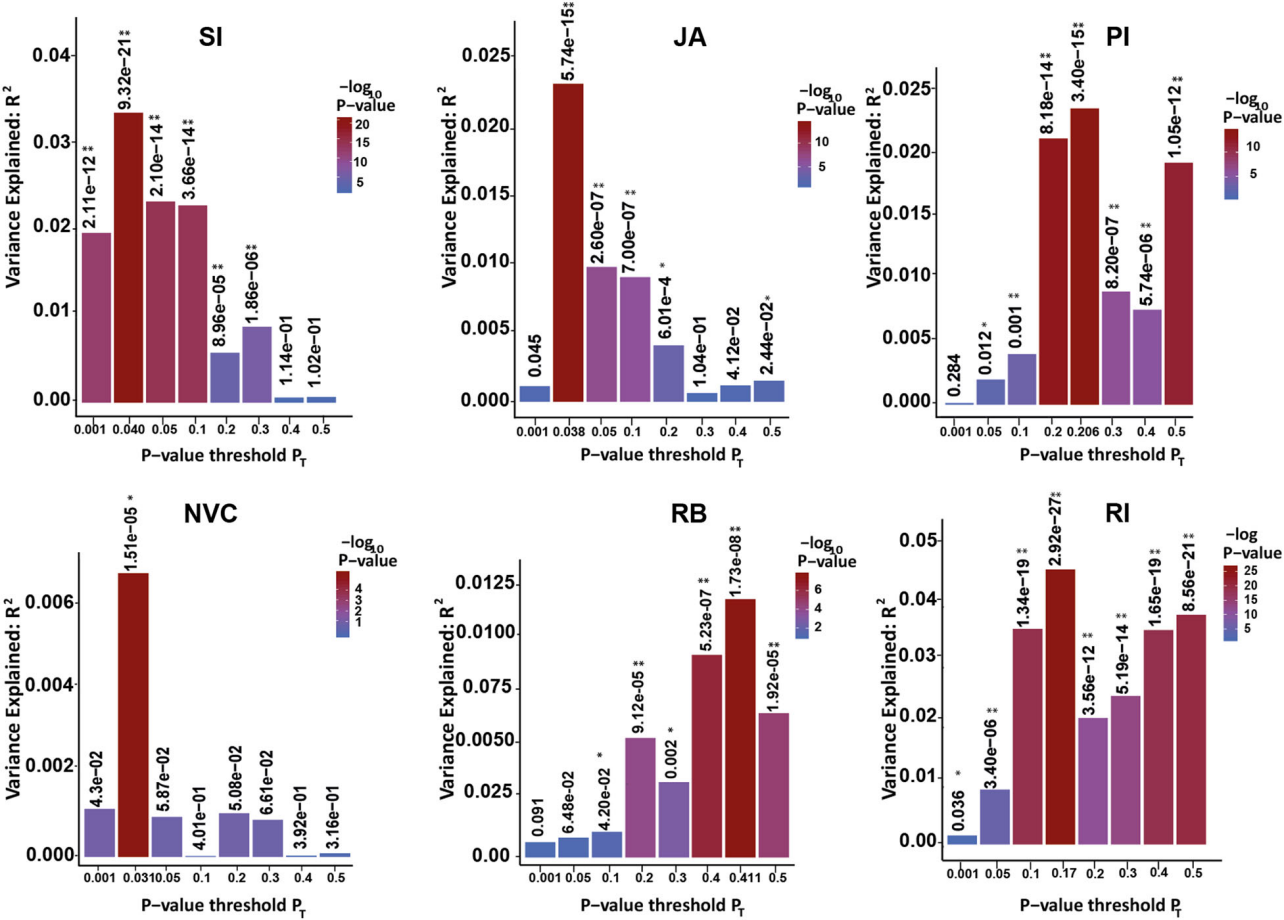
Polygenic risk score analysis showing the shared genetic etiology between ASD diagnosis and individual subdomains. Each bar represents the respective *P*-value thresholds (P_T_), whereas the numbers above bars denote the *P* value for the underlying model. SI social interaction, JA joint attention, PI peer interaction, NVC nonverbal communication, RB repetitive sensory-motor behavior, RI restricted interest.

The ASD–PRS explained a significant (all *P* < 2 × 10^−05^) proportion of genetic variance of all subdomains. The best PRS model explained 3.3% of variance (*R*²) in SI and 2.3% in JA and in PI. In contrast, *R*^2^ was lower for NVC (0.7%) and RB (1.2%), whereas for RI, the best model explained 4.5% of variance. *P*-value thresholds used for SNP selection of the subdomain GWAS in the best models ranged from 0.031 to 0.411 (Fig. 1).

#### Genetic heritability (*h*^2^_*SNP*_)

Significant *h*^2^_*SNP*_ (*P* < 0.05) was identified for all subdomains with the highest *h*^2^_*SNP*_ observed for SI (*h*^2^_*SNP*_ = 0.53, *P*_adjusted_ = 3.33 × 10^−16^), and the lowest for RB (*h*^2^_*SNP*_ = 0.21, *P*_adjusted_ = 6.72 × 10^−08^) (Supplementary Table 2).

#### Cross-trait correlations

**Fig. 2:**
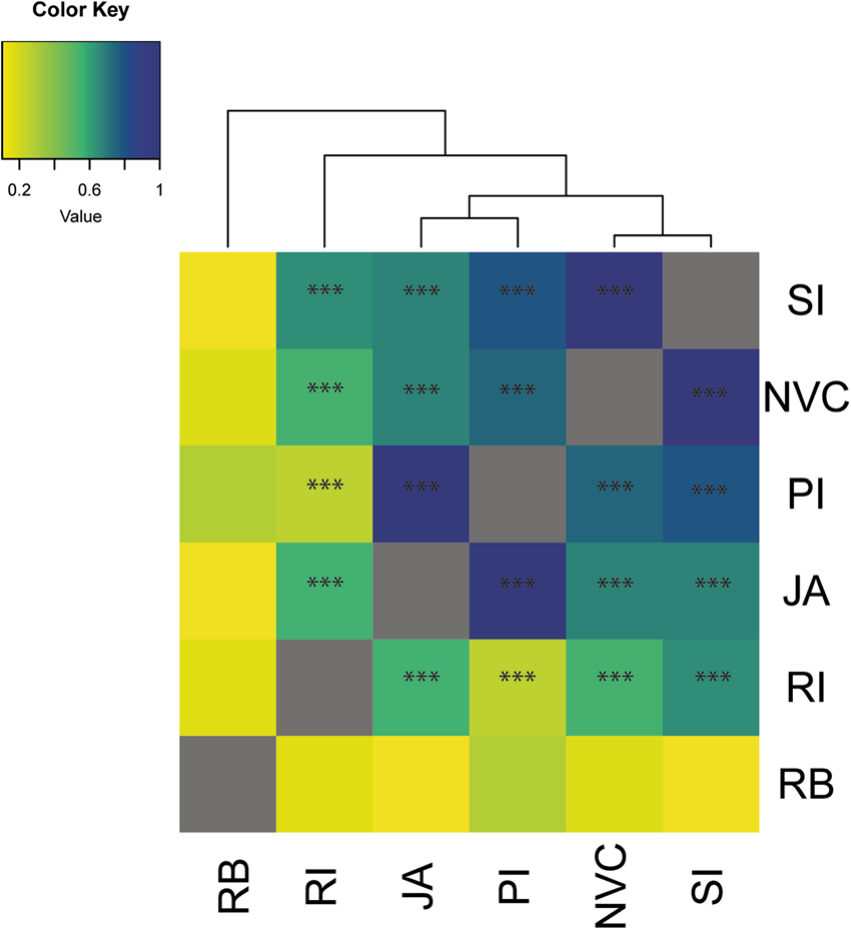
Genetic correlations (*r*_*g*_) among six subdomains based on the merged cohort. Asterisks mark significances with *** being corrected for *P* < 0.001. SI social interaction, JA joint attention, PI peer interaction, NVC nonverbal communication, RB repetitive sensory-motor behavior, RI restricted interest.

The strongest *r*_*g*_ was observed between SI and NVC (*r*_*g*_ = 0.97, *P* = 1.19 × 10^−11^). Moderate correlations were observed between SI and PI (*r*_*g*_ = 0.79, *P* = 2.19 × 10^−6^), SI and JA (*r*_*g*_ = 0.67, *P* = 7.47 × 10^−11^), and SI and RI (*r*_*g*_ = 0.64, *P* = 4.2 × 10^−7^), while the least correlation was observed between SI and RB (*r*_*g*_ = 0.10, *P* = 0.280). However, JA and PI were highly correlated (*r*_*g*_ = 1, *P* = 2.62 × 10^−10^). Moderate correlations were observed between JA and NVC (*r*_*g*_ = 0.66, *P* = 1.89 × 10^−5^) and RI (*r*_*g*_ = 0.55, *P* = 1.35 × 10^−4^). The lowest *r*_*g*_ with respect to JA was observed with RB (*r*_*g*_ = 0.11, *P* = 0.285). For PI, middle-range correlation was observed with SI (*r*_*g*_ = 0.79, *P* = 2.19 × 10^−6^), and NVC (*r*_*g*_ = 0.74, *P* = 4.78 × 10^−5^), whereas lower *r*_*g*_ values were seen for RB (*r*_*g*_ = 0.29, *P* = 0.127) and RI (*r*_*g*_ = 0.25, *P* = 0.077). Lowest *r*_*g*_ of NVC was observed with RB (*r*_*g*_ = 0.32, *P* = 0.040), whereas moderate *r*_*g*_ with RI (*r*_*g*_ = 0.68, *P* = 1.0 × 10^−4^). RB showed very low genetic correlation with RI (*r*_*g*_ = 0.15, *P* = 0.213). Overall, RB showed no significant *r*_*g*_, i.e., *P* < 0.05 with any other subdomain (Fig. 2).

#### Quantitative GWAS

**Fig. 3:**
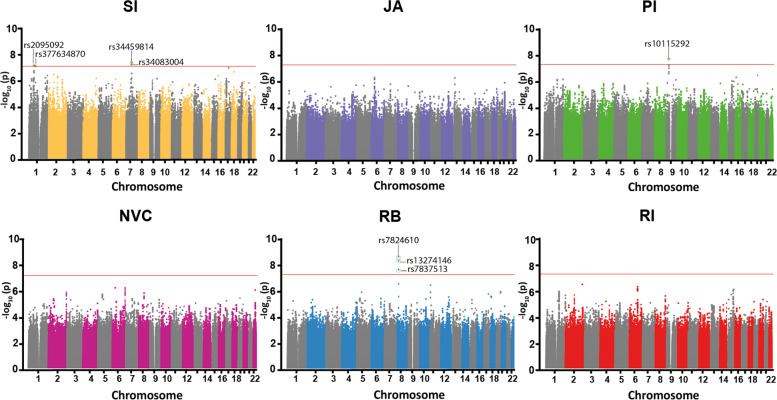
Manhattan plots of the six subdomains of the merged cohort. The *x* axis shows chromosome 1–22; the *y* axis shows the –log_10_*P* value, where each individual dot represents a SNP. The red line here shows the genome-wide significance threshold, i.e., *P* = 5 × 10^−8^ and the respective SNPs are mentioned.

GWAS (combined cohort) identified eight genome-wide significant SNPs (Fig. 3, Supplementary Fig. 4, Supplementary Tables 4, 5), which are reported along with their chromosomal position and closest gene as follows: four were found for SI, i.e., rs2095092, *P* = 4.3 × 10^−08^ at 1p31.3 (*PATJ*), rs377634870, *P* = 4.8 × 10^−08^ at 1p22.3 (no gene within 10 kb), rs34459814, *P* = 2.5 × 10^−08^ at 7q11.23 (*CLIP2*), rs34083004, *P* = 3.7 × 10^−08^ at 7q11.23 (*CLIP2*), one for PI, i.e., rs10115292, *P* = 1.8 × 10^−08^ at 9p21.1 (no gene within 10 kb), and three for RB, i.e., rs13274146, *P* = 2.1 × 10^−08^ at 8p21.3 (no gene within 10 kb), rs7837513, *P* = 4.2 × 10^−09^ at 8p21.3 (no gene within 10 kb), and rs7824610, *P* = 2.0 × 10^−09^ at 8q21.11 (no gene within 10 kb). No significant hit was identified for RI. For locus plots, see Supplementary Fig. 5.

### Gene-based analysis

**Fig. 4:**
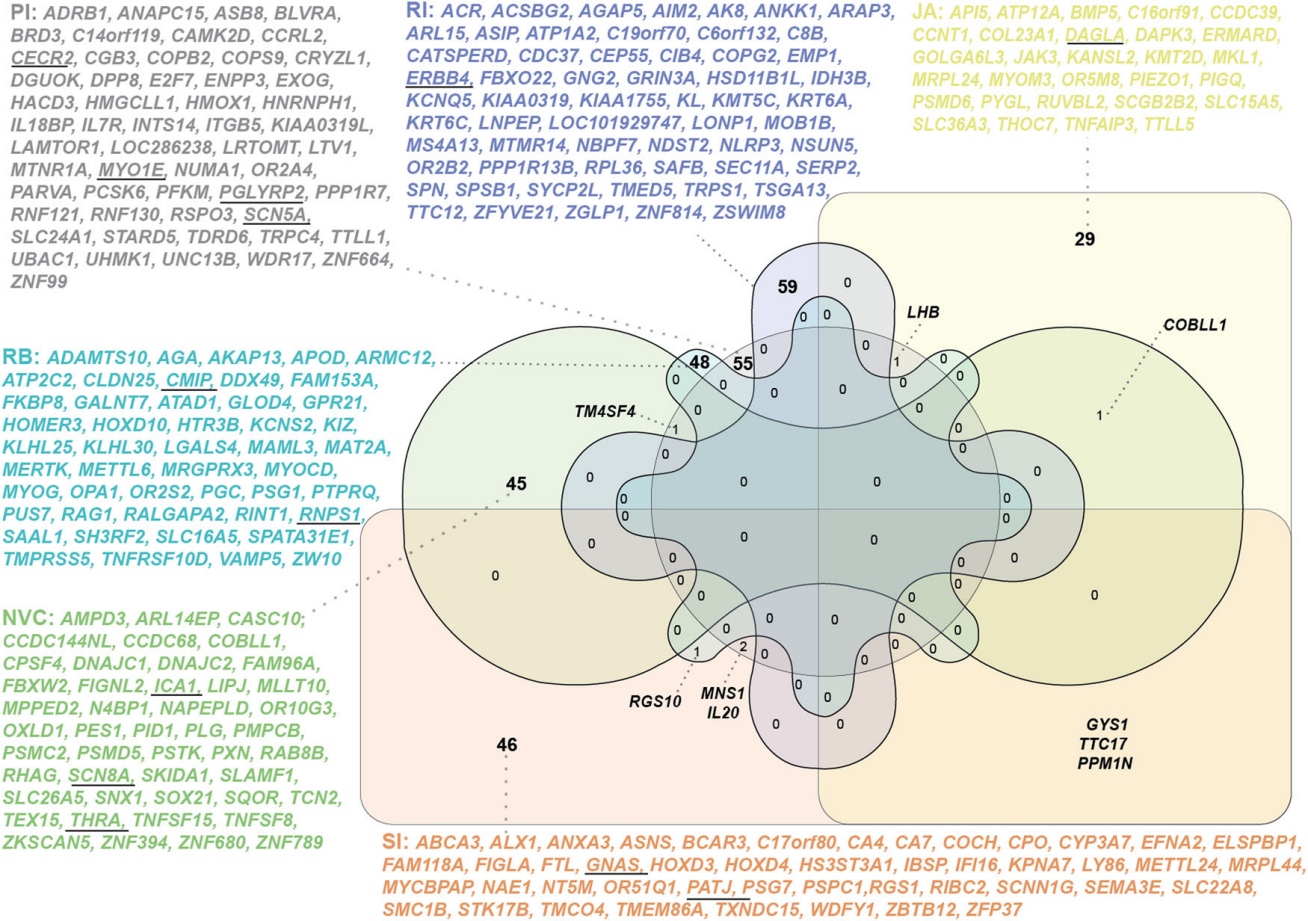
Venn diagram of overlapping genes with a significant *P*_permuted_ < 0.05 as identified using MAGMA from the individual cohorts, i.e., AGP and DE. The underlined genes represent SFARI genes. SI social interaction, JA joint attention, PI peer interaction, NVC nonverbal communication, RB repetitive sensory-motor behavior, RI restricted interest.

MAGMA identified 292 replicated (DE and AGP cohort *P*_permuted_ < 0.05) genes associated with any of the subdomains (Fig. 4; Supplementary Tables 6–8). The 52 associated genes with SI were enriched for GO terms, including “sensory perception”, and at brain level, the childhood-activated co-regulated brain gene-network module 6^37^ (beta = 3.213, *P* = 0.042, *P*_adj_ = 1). For JA, 35 genes were associated and nriched for GO terms, e.g., “carbohydrate and energy metabolism” and “chromatin modification”. For PI, 59 genes were identified, which are implicated in “hormone processing” and “plasma membrane” processes. For NVC, 47 genes were enriched for GO terms related to protein catabolism, and at brain level, the brain-expressed module 27 (beta = 3.297, *P* = 0.039, *P*_adj_ = 1) was enriched. The brain-enriched module *P* values were tested for multiple correction within the individual subdomain but not across subdomains. RB-associated 49 genes were enriched for “skeletal muscle tissue development”, “DNA binding”, and “transmembrane receptor activity”. For RI, 59 genes were identified and implicated in “postsynaptic” and “intracellular mediated” signaling along with regulation of MAPKKK (mitogen-activated protein kinase kinase kinase) cascade.

No genome-wide significant hit was overlapping between subdomains. However, 149 nominal (*P* < 0.01) SNPs were shared between SI, JA, and PI; 27 SNPs between SI, PI, and NVC. No nominal overlaps were identified between RI and any other phenotype. At gene level *P*_permuted_ < 0.05, we observed three overlapping genes between SI and JA *(GYS1, TTC17*, and *PPM1N*), two genes between SI and PI (*MNS1* and *IL20*), one gene between NVC and PI (*TM4SF4*), SI and RB (*RGS10*), JA and PI (*LHB*), and JA and NVC (*COBLL1*) (Fig. 4).

## Discussion

In this study, we studied common genetic variants for their role in shaping the phenotypic variability of ASD. We focused on ADI-R-derived phenotypic subdomains to determine their underlying genetic etiology and possible genetic and functional overlap. A large amount of variance was not explained by the PRS, implicating additional common and/or rare variation in the phenotypic expression of the subdomains. We also studied the *r*_*g*_ of individual subdomains and estimated the polygenetic risk for ASD to explore if variability in subdomains may be explained by general common genetic risk for ASD. Measures explaining phenotypic heterogeneity often have been studied as predictors of outcome in clinical trials^38^ or of long-term outcomes^39^, but genetic studies aiming at describing the genetic underpinnings of this phenotypic heterogeneity are scarce.

We identified and confirmed the six-factor structure of the ADI-R algorithm items first reported by Liu et al.^3^ in two independent ASD datasets. A similar six-factor solution has been published for 98 ADI-R algorithms^40^. Previously, another study conducted a factor analysis on 11 items related to restricted and repetitive behavior (RRB) and identified two factors, i.e., RSM and IS similar to our identified subdomains of RB and RI, respectively^10^. Thus, the identified subdomains in our study have been well replicated in independent ASD datasets before, and are plausible targets for quantitative genetic analyses.

*h*^2^_*SNP*_ has been studied in large ASD samples to quantify additive heritability explained by genome-wide SNPs^41^. Our study is the first estimating SNP-based heritability of specific phenotypic subdomains in ASD. We assumed that the heterogeneous phenotype of ASD may prevent a clear picture of the role of SNP in each subdomain. Overall, we observed low SNP-based heritability for the individual subdomains; however, in our study, *h*^2^_*SNP*_ for all subdomains was higher than previously reported estimates of the categorical phenotype ASD (~17%)^42^. Although, without replication, we cannot generalize our findings for the specific subdomains, we still describe higher heritability estimates for domain A-related subdomains. However, we observe a difference between the subdomains of domain B, which showed the lowest estimate for RB but higher estimates for RI. From the results of our study, we suggest a differential role of common and rare variants in domain A and also within the subdomains of domain B. This also may explain the lower SNP-based heritability of the categorical ASD phenotype, because it is defined by symptoms in domains A and B.

Phenotypic subdomains with high SNP-based heritability but lack of genome-wide significant hits, such as RI, might underlie many variants with low effect sizes. Subdomains with only little variance explained by SNP heritability where genome-wide hits are identified, might in contrast underlie few variants with a moderate-to-high effect size. Thus, we conclude that the genetic architecture underlying the phenotypic variance in ASD individuals is likely to be different across the domains.

The highest genetic correlation was identified between SI and NVC (0.97), mirroring the correlation at the phenotypic level^43^. A complete genetic correlation of 1 was found for JA and PI, suggesting strongly overlapping common genetic variation underlying SI and NVC, or JA and PI. Moreover, we observed that subdomains of domain A are also highly phenotypically correlated than the subdomains of domain B (Supplementary Fig. 6). In contrast to SNP-based heritability, genetic correlation analysis of the two subdomains related to domain B showed only weak correlation, and thus may be genetically independent with respect to common variation. Another linkage study on ADI-R algorithm-derived “repetitive sensory-motor behavior” (RSMB) and “insistence on sameness” (IS) scores^44^ similarly reported predominantly specific, but also a few overlapping linkage findings for these subdomains. A recent qGWAS reported suggestive evidence for distinct common variants when RSMB and IS were analyzed independently; however, when both phenotypes were considered together, three genome-wide hits were identified^10^. This indicates higher variability of the combined phenotypic measure, resulting in a higher power to detect a specific genetic risk. RB did not genetically correlate with other subdomains and also had the lowest *h*^2^_*SNP*_ (0.21). Similarly, a population-based twin study did not find genetic covariation between SI and RB scores^45^; however, a twin study of ASD individuals reported a strong genetic overlap of the extreme values of impaired social communication and restricted behaviors derived from SCQ^46^. The contrasting findings may be explained by a differential role of common and rare variation in social communication-related subdomains and RB, especially in ASD individuals, with rare variation playing a stronger role in RB^47^.

With respect to specific genetic variation underlying the different subdomains, several genome-wide significant hits and novel candidate genes were identified in the present study. For SI, we observed an association with *PATJ* (aka *INADL*) at SNP as well as at gene level. *PATJ* is coding for a scaffolding protein CIPP, and regulates surface expression of the acid-sensing ion channel 3 in sensory neurons^48^. The Uniprot Protein Database (https://www.uniprot.org/) predicts, based on sequence similarities, an interaction of *PATJ* with glutamatergic NMDA receptors and ASD candidate genes *NLGN2* and *HTR2A*. Rare loss-of-function variants in *PATJ* have also previously been found in ASD^49^, thus strengthening our findings. The second genome-wide significant hit for SI mapped to *CLIP2* gene is located at 7q11.23. Duplication carriers of this region show a high rate of ASD^50,51^. Furthermore, SI-associated genes were enriched in a co-expressed brain gene set (module 6). This module is mainly active in cortical structures during early childhood. In the hippocampus, module 6 is activated before birth, silenced prior to puberty, and then reactivated. This supports previous findings of early cortical maturation impairments in ASD^52^, and of the important role of the hippocampus in social behavior^53^.

No genome-wide hit was identified for JA. At the gene level, JA was associated with *DAGLA* gene implicated in seizures and neurodevelopmental disorders, including autism^54^, and the *COBLL1* gene involved in epilepsy^55^ and language impairment^56^.

The only genome-wide significant SNP in PI is rs10115292 mapped to an intergenic region at chr. 9p21.1, known for ASD-associated CNVs^57^. Among the significant genes (*P*_permuted_ < 0.05) enriched for PI, we identified a sodium voltage-gated ion channel gene *SCN5A* that was found to be a hub protein in an ASD-associated protein-interaction module^58^. Other ASD-associated significant PI genes include *CECR2*, a 7.2-kb exonic loss, which was found in an ASD female^54^.

For NVC, no genome-wide significant hit was identified. Most suggestively associated SNPs map to chr. 6q26, a region linked to ASD^59^. *SLC26A5* at 11p15.4 was among the top hits from the gene-based analysis; mutations in this gene are potential candidates for causing neurosensory deafness^60^. This region is linked with delayed development of speech^61^. The NVC-associated regulatory gene set (module 27) is expressed in the hippocampus, striatum, and mediodorsal nucleus of the thalamus until puberty (Supplementary Fig. 7). These regions are well known for their role in language and communication^62,63^, which puts our findings in line with the current literature.

RB was associated with genome-wide significant SNPs at 8p21.3, a region previously associated with restricted and repetitive behaviors in ASD^10^. Duplications of this region have been associated with ASD^64^. The suggestive effect at 19q13.33 is also in line with previous findings regarding RB^3^. Gene-based analysis indicated *RGS10* gene implicated in neurodegenerative diseases^65^, and is also overlapping in SI and RB.

Top significant SNP hits for RI were also observed in migraine, sensorineural deafness, cognition, Williams–Beuren syndrome, and ASD such as *NLPR3*^66^, *GNG2*^67^, and *NSUN5*^68^. No genome-wide associated SNP was identified for RI. The top peak at 15q25.3, however, is spanning the *NTRK3* gene, associated with autism and Asperger syndrome^69^, as well as obsessive–compulsive disorder^70^.

Among the overlapping genes in the subdomains, we identified *GYS1* in JA and SI. KO of Gys1 has been known to induce depression-like behavior in rats, indicating that brain glycogen has an important role in animal emotion^71^. Another study generated a brain-specific GYS1 KO mouse and found that these animals had a significant deficiency in motor and cognitive abilities and synaptic strength^72^. Another overlapping gene found between JA and NVC is *COBLL1*. A study reported an individual with ASD and Tourette syndrome with heterozygous microdeletion of approximately 719 kb at 2q24.3, which led to deletion of *COBLL1* gene as well besides four other genes. As mentioned above, this gene is also found to be deleted in a patient with severe epilepsy^55^ and individuals with autistic features, developmental delay, repetitive hand movements, and language impairments^56^.

One of the major limitations of our study is the limited sample size of individual AGP and DE cohorts. Although quantitative statistical tests generally have a higher power in comparison with the qualitative approaches, small effects are likely to have been undetected in our study (see power analysis in the methods section). It is possible that a variant may carry a large genetic risk to increase expression of one phenotypic subdomain but a smaller risk on another. Thus, to identify the overlapping SN-based genetic risk with high confidence, it requires a larger sample size to attain an adequate statistical power. However, we followed a conservative approach to minimize proneness of false positives by performing the gene analysis in two independent ASD datasets, and to classify genes as replicated only if they have an empirical *P* < 0.05 in both cohorts. Although this cannot omit the possibility of false positives and especially not false-negative findings, it lowers the risk for false findings.

Another limitation of our study is the mixed ethnicity in the two cohorts and higher ASD severity scores in the AGP sample. However, we accounted for the mixed ethnicity in our GWAS analysis, and to overcome false-positive associations, we followed a conservative approach by performing gene-based analysis in two independent ASD datasets and only interpreted overlapping hits. In addition, several genes mapped from GWAS hits of the combined cohort were found at gene level. For the PRS analysis, we used the combined cohort, which contained PGC ids as well, but since our research question was focused on dimensional phenotypes rather than categorical, so we did not exclude those samples from our cohort. In the heritability and genetic correlation analysis, we did not account for covariates. This might have led to an overestimation of estimates. Still, a recent study has shown that the inclusion of covariates can result in inflated and biased genetic correlations and heritability estimates^73^. Thus, we again chose the more conservative approach. However, we suggest replicating the analysis in a genetically more homogeneous sample.

In summary, our results suggest that the genetic architecture of subdomains is distinct between A- and B-related subdomains and differs within the two B-related subdomains RB and RI. We replicated several previously implicated genes in ASD, but also describe new candidate genes for specific subdomains. Involved biological pathways and gene expression patterns strengthen the previous observations that ASD phenotypic variability is influenced by pathways regulating neuronal development of different brain areas, including the hippocampus, amygdala, and cortical areas.

The results of our study need to be replicated in larger samples with different ethnic backgrounds. In addition, a combined analysis of common and rare variants may clarify the specific role of common variants in shaping the ASD phenotype in relation to the reported subdomains.

## Supplementary information

Supplementary Material file

Supplementary Table 2

Supplementary Table 6

Supplementary Table 7

Supplementary Table 8
