# Supplementary material for: Quantitative genome-wide association study of six phenotypic subdomains identifies novel genome-wide significant variants in autism spectrum disorder
Source: Transl Psychiatry. 2020 Jul 5;10:215. doi: 10.1038/s41398-020-00906-2 (PMC7335742; doi:10.1038/s41398-020-00906-2)
Supplement: Supplementary file 1 — Supplementary Material file [file 41398_2020_906_MOESM1_ESM.docx]

**Supplementary material**

# Quantitative genome-wide association study of six phenotypic subdomains identifies novel genome-wide significant variants in Autism Spectrum Disorder

**Running title:** Phenotypic subdomains GWAS in ASD

Afsheen Yousaf^1^, Regina Waltes^1^, Denise Haslinger^1^, Sabine M Klauck^2^, Eftichia Duketis^1^, Michael Sachse^1^, Annette Voran^3^, Monica Biscaldi^4^, Martin Schulte-Rüther^5-6^, Sven Cichon^7-10^, Markus Nöthen^10^, Jörg Ackermann^11^, Ina Koch^11^, Christine M. Freitag^1 *^, Andreas G. Chiocchetti^1 *^

^1^Department of Child and Adolescent Psychiatry, Psychosomatics and Psychotherapy, University Hospital Frankfurt, Goethe University, Frankfurt am Main, Germany

^2^Division of Molecular Genome Analysis and Division of Cancer Genome Research, German Cancer Research Center (DKFZ), Heidelberg, Germany

^3^Department of Child and Adolescent Psychiatry, Saarland University, Homburg, Germany.

^4^Department of Child and Adolescent Psychiatry, University Hospital Freiburg, Freiburg, Germany

^5^Translational Brain Medicine, Department of Child and Adolescent Psychiatry, Psychosomatics, and Psychotherapy, RWTH Aachen University, Aachen, Germany

^6^JARA-BRAIN, Aachen, Germany

^7^Institute of Neuroscience and Medicine (INM-1), Research Center Juelich, Juelich, Germany

^8^Human Genomics Research Group and Division of Medical Genetics, Department of Biomedicine, University of Basel, Basel, Switzerland

^9^Institute of Medical Genetics and Pathology, University Hospital Basel, Basel, Switzerland

^10^Department of Genomics, University of Bonn, Bonn, Germany

^11^Molecular Bioinformatics, Institute of Computer Science, Johann Wolfgang Goethe-University Frankfurt am Main, Frankfurt am Main, Germany

* shared last authors

**Corresponding author**

Name: Afsheen Yousaf

Department: Child and Adolescent Psychiatry, Psychosomatics and Psychotherapy, Frankfurt am Main Germany

**Index**

**Supplementary Material and Methods** 4

***Sample description*: 4**

***Genotype data:* 5**

Quality Control (QC) and Imputation 5

***Power analysis***: 6

Heritability and genetic correlation 6

Quantitative GWAS 6

***Phenotype data:*** 6

ADI-R algorithm items and phenotype imputation 6

Principal component analysis 6

***Single Nucleotide Polymorphisms (SNPs) based analysis:*** 7

Genetic heritability 7

Genetic Correlation 7

Polygenic Risk Scores (PRS) 8

***Gene-wise analysis:*** 8

Gene-wise analysis of GWAS summary statistics and GO term enrichment analysis: 8

Network analysis 8

**Supplementary tables 10**

Supplementary Table 1: Sample adequacy test for AGP cohort 10

Supplementary Table 2: Factor loadings and heritability estimates 10

Supplementary Table 3: Confirmatory factor analysis in DE cohort 10

Supplementary Table 4: Genome-wide significant SNPs in Combined, AGP and DE cohorts 11

Supplementary Table 5: MAGMA genes associated with the subdomains 13

Supplementary Table 6: GO pathways enriched for the subdomains 13

Supplementary Table 7: Enrichment output Kang modules for the subdomains 13

Supplementary Table 8: Lambda estimates of each subdomain for the combined cohort 13

**Supplementary figures 14**

Supplementary Figure 1 14

Supplementary Figure 2 15

Supplementary Figure 3 16

Supplementary Figure 4 17

Supplementary Figure 5 23

Supplementary Figure 6 24

Supplementary Figure 7 25

References 26

**Supplementary Material and Methods**

## *Sample description:*

Two large ASD cohorts, a German cohort (DE), and the Autism Genome Project (AGP) cohort were included. German patients were recruited at the Departments of Child and Adolescent Psychiatry at Goethe-University Frankfurt am Main, University Hospital Bern, Saarland University Hospital, and University Hospital Freiburg ^1^. All participants or caregivers provided written informed consent. The local ethical committees approved the study (decisions 162/99 (Frankfurt); 214/10 (Bern); 73/04 (Homburg), 237/09 (Frankfurt)). ICD-10 diagnostic criteria of autism (F84.0), Asperger’s disorder (F84.5), or atypical autism (F84.1) were ensured by experienced child psychiatrists or clinical child psychologists, based on a medical and developmental history, direct observation, the Social Communication Questionnaire SCQ ^2^; the Autism Diagnostic Interview-Revised (ADI-R) ^3,4^ and/or the Autism Diagnostic Observation Schedule (ADOS) ^5,6^. We excluded all individuals with schizophrenia, bipolar disorder, a neurodegenerative disorder, a known cytogenetic finding, fragile-X Syndrome, Angelman syndrome, Prader-Willi syndrome, Rett syndrome or if there was any other genetic disorder diagnosed. Also individuals with IQ<35, history of a severe medical condition, birth weight <1500 grams or cerebral palsy were excluded. A detailed description of the clinical assessment is provided in^7,8^. The Autism Genome Project (AGP) cohort included n=2,730 trio families and n=5 parent-child duos collected at 15 clinical sites across the US, Canada, and Europe. Overlapping German samples were excluded from the AGP cohort for the present analysis. Diagnostic criteria followed DSM-III-R (Diagnostic and Statistical Manual of Mental Disorders-Revised) or DSM-IV-TR (Diagnostic and Statistical Manual of Mental Disorders Text Revised) for autism, Asperger’s disorder and Pervasive Developmental Disorder supported by the Autism Diagnostic Interview-Revised (ADI-R) and/or Autism Diagnostic Observation Schedule (ADOS). Exclusion criteria and sample quality checks for AGP data were the same as for the German data. Further details on the AGP data are published in the work of Anney et al ^9^. For the final analysis, only one affected individual per family was included i.e. the index patient.

## *Genotype data:*

### Quality Control (QC) and Imputation

QC and imputation were performed on AGP and DE cohorts separately based on standardized protocols ^10^using an *in-house* developed tool MAGNET^11^, the QC part is mostly implemented in *Plink 1.9*^12^. In short: SNPs and individuals with >5% missing rate were excluded. Samples i) discordant between genetic and database gender, ii) with large chromosomal aberrations/ abnormalities iii) with an inbreeding coefficient (F) > 0.2 (contamination) and F < -0.15 (inbreeding) and iv) with >1% of Mendel errors were removed. Variants with Hardy-Weinberg Equilibrium *P*< 10^-8^, Mendel errors > 10% and a Minor Allele Frequency (MAF) < 2% were excluded. Four genetic components (C1 to C4) reflecting population stratification were extracted using plink v1.9^12^ (Supplementary Figure 1). Criteria are based on the Ricopili^10^ pipeline and the standards applied by the ASC consortium^13^. Population stratification was also performed in *Plink* which uses complete linkage agglomerative clustering based on identity by state (IBS) distance and identifies pairs of individuals with $\hat{\pi}$ (PI-HAT: measure for IBS) > 0.2, one subject of each pair is excluded. Cluster analysis is performed based on the pre-computed IBS followed by a multidimensional scaling analysis on the genome-wide IBS pairwise distances. A multidimensional plot is generated representing the first two components and the first four dimensions are extracted for further analysis.

Before performing the genome-wide imputation of SNPs, we ensured that all SNP names and annotated genomic locations for the genotype data matched with the genome build of reference alleles i.e. 1000 Genomes phase 3 dataset ^14^. Imputation was performed separately in each cohort. The 1000 Genomes phase 3 data is annotated with GRCh37 (Genome Reference Consortium Human build 37) coordinates. Genetic sample data not corresponding to GRCh37 annotation were converted using batch coordinate conversion program *liftOver* ^15^. SNPs which were in the provided genotype data and not in the reference data were removed. Phasing was performed using *SHAPEIT* ^16^. SNP inconsistencies such as strand flip issues that cannot be resolved by flipping were removed. In the final step, imputation was performed using Minimac3 ^17^. Imputation output is filtered based on quality of imputation scores with Rsq > 0.3 (removes > 70% of poorly-imputed SNPs at the cost of <0.5 % well-imputed SNPs) ^18^. At the end of the imputation process, we again run the QC pipeline on imputed genotype data to filter the SNPs falling below the QC thresholds. After quality control and imputation, we ended up with 6,900,500 SNPs overlapping in both cohorts.

### *Power analysis:*

#### Heritability and genetic correlation

Power analysis (GTCA-calculator) indicated that to attain a power of 1-beta > 80% assuming a *h^2^SNP* similar to the diagnosis of ASD of *h^2^SNP* 0.4, a sample size of n> 2,241 is needed^19^. The final combined AGP and DE cohorts included n= 2,509 individuals.

#### Quantitative GWAS

The sample size had a power of 1-beta > 80% to explain 6% of the variance (R²=0.06) in the DE cohort, 1.5% in the AGP cohort and 1.2% in the combined cohort with a genome-wide significance threshold of alpha = 5e^-8^. Power analysis was performed using Quanto (<http://biostats.usc.edu/Quanto.html>).

## *Phenotype data:*

### ADI-R algorithm items and phenotype imputation

Based on the study by Liu et al., 2011^20^ we selected 28 “ever/most abnormal” items from the ADI-R diagnostic algorithm available for both verbal and non-verbal individuals. These 28 items are shown in Supplementary Table 2. For each item, the highest value between “ever” or “most abnormal” scores were selected. ADI-R diagnostic algorithm scores of 3 were recoded as 2 to limit the impact of severity in the overall diagnostic process. These 28 item values were then used for further factor analysis. Individuals who had >10% missing items (i.e. n=3) were excluded; for all other individuals, the missing values were imputed.

The 28 ADI-R items from the AGP and German dataset were combined for phenotype imputation. Multiple imputation by chained equations implemented in R package mice was done applying predictive mean matching (pmm) ^21^. We implemented 10 cycles of imputations as suggested per the rate of missing data ≤ 10% ^22,23^. We then performed sample adequacy tests for AGP data before performing principal component analysis.

### Principal component analysis

Principal Components Analysis (PCA) was performed with varimax rotation on 28 ADI-R items using the R package psych ^24^ as in previous studies ^25,26^. PCA was preferred over the classical exploratory factor analysis (EFA) approaches to preserve the maximum amount of variation and independence in the resulting factors which can play a role in determining that the genetic factors might be independent of each other. Whereas, EFA does not account for the maximum variation and estimates interdependence between variables to find common factors, which might not necessarily be independent of each other. Orthogonal rotation was performed to minimize the number of variables with high loadings on each factor.

The best factor solution was identified based on Kaiser’s criterion ^27^ and scree plot ^28^. Factors were retained if (i) each factor have at least three items loaded onto it ^29,30^, however, additional variables improve factor stability ^31^; (ii) factor loadings of respective items >0.4, which is stringent than otherwise defined criteria (>0.35) ^32^; and (iii) factors are interpretable. The respective items were summed up to provide a combined score which will be used for subsequent analysis.

## *Single Nucleotide Polymorphisms (SNPs) based analysis:*

The following analyses were based on the combined dataset (DE and AGP) to account for the power issues. For calculating genetic heritability we did not correct for covariates since it has been shown that inclusion of covariates in heritability and genetic correlation analysis can bias the estimates for restricted maximum likelihood (REML). Moreover, the inclusion of principal components (PCs) of divergent populations as covariates can result in inflated and unstable genetic correlation^33^.

### Genetic heritability

SNP based heritability for each ADI-R algorithm derived subscore was estimated by the tool GCTA ^34^ since the sample size was not adequate for using LDSC^35^. The variance explained by genome-wide SNPs (i.e., variance explained by all the causal variants) is estimated based on two steps i.e. (i) a genetic relationship matrix (GRM) calculated for all SNPs. This is an *N x N* matrix where each element represents the genetic similarity of two individuals. (ii) The second step performs REML analysis where GRM is used as a predictor in the mixed linear model with the individual subdomain as the dependent variable ^36^.

### Genetic Correlation

The genetic correlations are calculated using bivariate GCTA–GREML from *GCTA* tool ^34^ based on the merged cohort containing the phenotypic and genotypic information from unrelated individuals. GCTA bivariate method is an extension of the univariate model which relates the pairwise genetic similarity matrix to a phenotypic covariance matrix between the first trait of interest with the second trait of interest and allows for correlated residuals ^37^.

### Polygenic Risk Scores (PRS)

To identify the level of shared genetic etiology between ASD and the ADI-R algorithm derived subdomains, we performed a polygenic risk score-based analysis using *PRSice*^38^ on GWAS summary data for both base and target datasets. Here the target dataset is the summary statistic output from GWAS of individual subdomains (i.e. JA, SI, PI, NVC, RB, and RI) in the combined cohort and the base dataset is the summary statistics data gathered from GWAS of ASDs by the Psychiatric Genomics Consortium (PGC) autism group (PGC-ASD GWAS, data for 5305 ASD cases and 5305 controls—Data is publicly available: <http://www.med.unc.edu/pgc/downloads>). Clumping was performed in *Plink* to attain SNPs that are largely independent of each other for LD with an LD threshold of r^2^>0.1 and a distance of 250 kb. PRS on the base phenotype are calculated, using GWAS results, in individuals from the combined data set, and these are used as predictors of the target phenotype in a regression. PRSice then calculates the sum of alleles which are associated with a trait of interest (e.g. SI in our analysis) weighted by their effect sizes estimated from the PGC-ASD GWAS, from which only the SNPs above the seven broad P-value thresholds (indicated by P_T_) were included, that is, the SNPs with P_T_<0.001, 0.05, 0.1, 0.2, 0.3, 0.4 and 0.5.

## *Gene-wise analysis:*

### Gene-wise analysis of GWAS summary statistics and GO term enrichment analysis

We used Multi-marker Analysis of GenoMic Annotation algorithm *MAGMA* v1.06 ^39^ to consider associations across a gene rather than each marker individually and calculated empirical p-values. The algorithm requires summary statistics (p-values) from GWAS. SNPs were mapped to corresponding genes within a window of 5 kilobases (kb) up and downstream of the respective hg19 annotated coding sequence. The significant genes (gene-wise empirical *P*-value < 0.05) identified in both cohorts were then subjected to Gene Ontology (GO) and pathway analysis using *GO-Elite* ^40^. The gene universe selected for performing the GO enrichment contained a total of 18,177 genes, i.e. all genes mapped using MAGMA tool.

### Network analysis

The significant overlapping genes from MAGMA analysis in both cohorts for each trait were selected to find the spatiotemporal pattern of these genes in a developing human brain. We used the human brain transcriptome dataset by Kang et al., ^41^ which is available to be downloaded from Allen brain atlas (<http://www.brainspan.org/>). This data covers transcriptome profiles of 16 different brain regions within a time frame ranging from embryonic development to late adulthood of males and females and consists of a total 1,340 tissue samples collected from one or both hemispheres of 57 post-mortem human brains. Co-expression pattern of genes within this dataset has shown to associate into 29 co-regulated gene modules that have been published in the original publication. For each of the 29 modules, the gene lists were kindly provided by the authors. We looked for enrichment of these significantly overlapping genes using Fisher’s exact test (not corrected for multiple comparisons) in the 29 gene modules representing specific spatiotemporal patterns. For enrichment analysis we used all the MAGMA as well as genes used in Kang et al., expression data set (Affymetrix GeneChip Human Exon 1.0 ST Array platform). 2D heatmaps with brain anatomical structures over time were plotted using the R package CerebroViz ^42^ to visualize the eigen-gene values for each module. Moreover, we plotted the gene networks of the modules including the top 50 connected module-genes and highlighted the genes implicated in the ADI-R factors tested using the R package igraph ^43^.

# Supplementary tables

## **Supplementary Table 1**: Sample adequacy test for AGP cohort

| **Sample Adequacy Test** | **Value** | **Cut-off value** |
| --- | --- | --- |
| Bartlett’ test of sphericity *P* -value | <0.0001 | <0.05 |
| Kaiser Maier Olkin | 0.92 | >0.5 |

## **Supplementary Table 2**: Factor loadings and heritability estimates of the six subdomains

(Provided as an excel table)

## **Supplementary Table 3:** Confirmatory factor analysis in DE cohort

| **Measure** | **Value** | **Cut-off for good fit** |
| --- | --- | --- |
| TLI (Tucker-Lewis Index) | 0.981 | >= 0.95 good model fit^44^ |
| CFI (Comparative Fit Index) | 0.983 | 0.95 (great); 0.90 traditional^44^ |
| RMSEA (Root Mean Square Error of Approximation): | 0.039 | < 0.08^45^ |
| SRMR (Standardized Root Mean Square Residual) | 0.048 | <0.09 good^44^ |

## **Supplementary Table 4: Genome-wide significant SNPs in Combined, AGP and DE cohorts**

|  | **Pheno.** | **SNP** | **CHR** | **Gene** | **Comb. beta** | **Comb.**  **Pval** | **AGP_beta** | **AGP_Pval** | **AGP_beta**  **(incl. IQ as covariate)** | **AGP_Pval**  **(incl. IQ as covariate)** | **DE_beta** | **DE_Pval** |
| --- | --- | --- | --- | --- | --- | --- | --- | --- | --- | --- | --- | --- |
| **Combined Cohort** | **SI** | rs2095092 | 1 | *PATJ* | -0.530 | *4.39e-08* | -0.466 | 2.39e-05 |  | - | -0.704 | 4.10e-04 |
|  |  | rs377634870 | 1 |  | 0.532 | *4.85e-08* | 0.578 | *1.58e-08* |  | - | 0.165 | 5.57e-01 |
|  |  | rs34459814 | 7 | *CLIP2* | 0.492 | *2.50e-08* | 0.488 | 1.86e-07 |  | - | 0.461 | 5.87e-02 |
|  |  | rs34083004 | 7 | *CLIP2* | 0.488 | *3.75e-08* | 0.483 | 2.90e-07 |  | - | 0.461 | 5.87e-02 |
|  | **PI** | rs10115292 | 9 |  | 0.388 | *1.83e-08* | 0.361 | *4.73e-08* |  | - | 0.406 | 8.88e-02 |
|  | **RB** | rs13274146 | 8 |  | -0.733 | *2.15e-08* | -0.733 | 9.44e-07 |  | - | -0.731 | 6.5e-03 |
|  |  | rs7837513 | 8 |  | -0.776 | *4.23e-09* | -0.783 | 2.28e-07 |  | - | -0.753 | 5.27e-03 |
|  |  | rs7824610 | 8 |  | -0.790 | *2.00e-09* | -0.806 | 1.00e-07 |  | - | -0.743 | 5.40e-03 |
| **AGP** | **SI** | rs377634870 | 1 |  | 0.532 | *4.86e-08* | 0.578 | *1.58e-08* | 0.5295 | 1.23e-07 | 0.165 | 5.57e-01 |
|  |  | rs9333127 | 10 | *ITGA8* | 0.414 | 2.81e-06 | 0.556 | *1.74e-08* | 0.5213 | 6.55e-08 | -0.092 | 6.36e-01 |
|  | **PI** | rs7777015 | 7 |  | 0.321 | 4.28e-06 | 0.447 | *4.75e-09* | 0.4063 | 9.08e-08 | -0.119 | 4.4e-01 |
|  |  | rs6963792 | 7 |  | 0.299 | 2.73e-05 | 0.453 | *7.89e-09* | 0.413 | 1.30e-07 | -0.201 | 1.99e-01 |
|  |  | rs7783341 | 7 |  | 0.303 | 2.34e-05 | 0.466 | *3.25e-09* | 0.426 | 5.69e-08 | -0.228 | 1.44e-01 |
|  |  | rs9969152 | 7 |  | 0.316 | 8.07e-06 | 0.470 | *1.65e-09* | 0.4366 | 1.79e-08 | -0.186 | 2.34e-01 |
|  |  | rs10115292 | 9 |  | 0.388 | 1.82e-08 | 0.361 | *4.73e-08* | 0.3342 | 1.36e-06 | 0.406 | 8.88e-02 |
|  | **RB** | rs441459 | 11 | *SLC22A18AS* | -0.435 | 1.21e-06 | --0.567 | *4.53e-08* | -0.5045 | 4.06e-07 | -0.089 | 6.19e-01 |
|  |  | rs388190 | 11 | *SLC22A18AS* | -0.438 | 3.16e-07 | --0.553 | *1.44e-08* | -0.5008 | 9.18e-08 | -0.099 | 5.76e-01 |
| **DE** | **JA** | rs2151874 | 1 |  | -0.495 | 6.63e-05 | -0.160 | 2.40e-01 |  | - | -1.600 | *1.95e-09* |

Genome-wide hits for combined and individual cohorts, italics show the P-values for genome-wide hits. Pheno. : Phenotype; Comb. :Combined; SI: social Interaction; JA: Joint Attention; PI: Peer Interaction; NVC: Non-verbal Communication; RB: Repetitive sensory-motor Behavior; RI: Restricted Interest. Only genome-wide hits in the combined cohort were reported.

## **Supplementary Table 5: Lambda estimates of each subdomain for the combined cohort**

| Subdomains  (Merged cohort) | Lambda |
| --- | --- |
| JA | 1.043 |
| SI | 1.057 |
| PI | 1.060 |
| NVC | 1.039 |
| RB | 1.032 |
| RI | 1.023 |

## **Supplementary Table 6: MAGMA genes associated with the subdomains**

(Provided as an excel table)

## **Supplementary Table 7: GO pathways enriched for the subdomains**

(Provided as an excel table)

## **Supplementary Table 8: Enrichment output Kang modules for the subdomains**

(Provided as an excel table)

# Supplementary figures

## **Supplementary Figure 1**


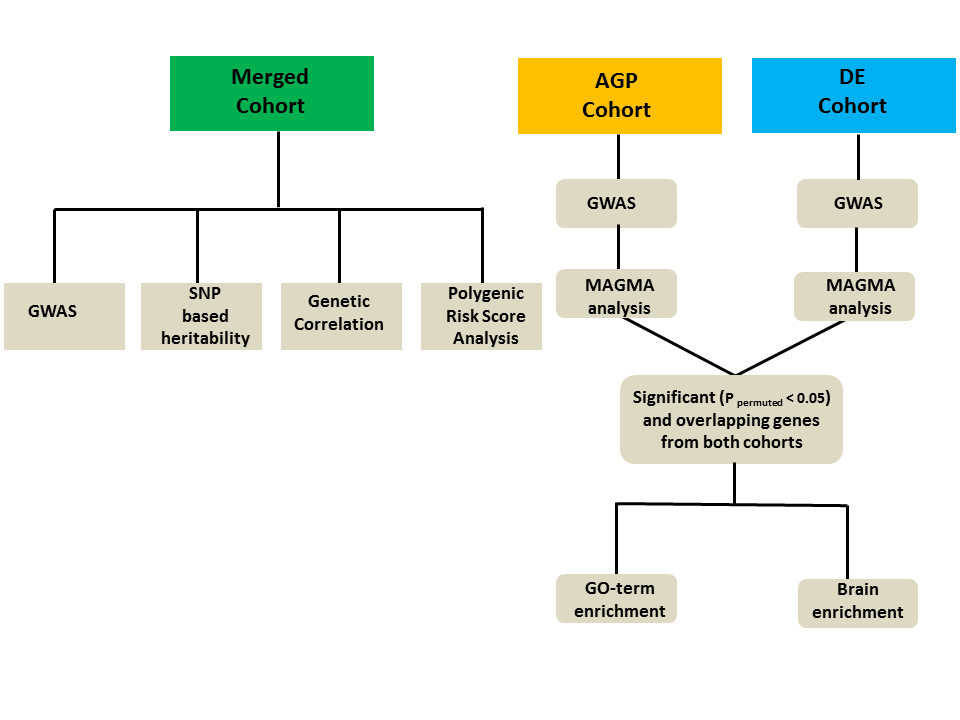


**Flowchart of the analysis perfomed**

## **Supplementary Figure 2**


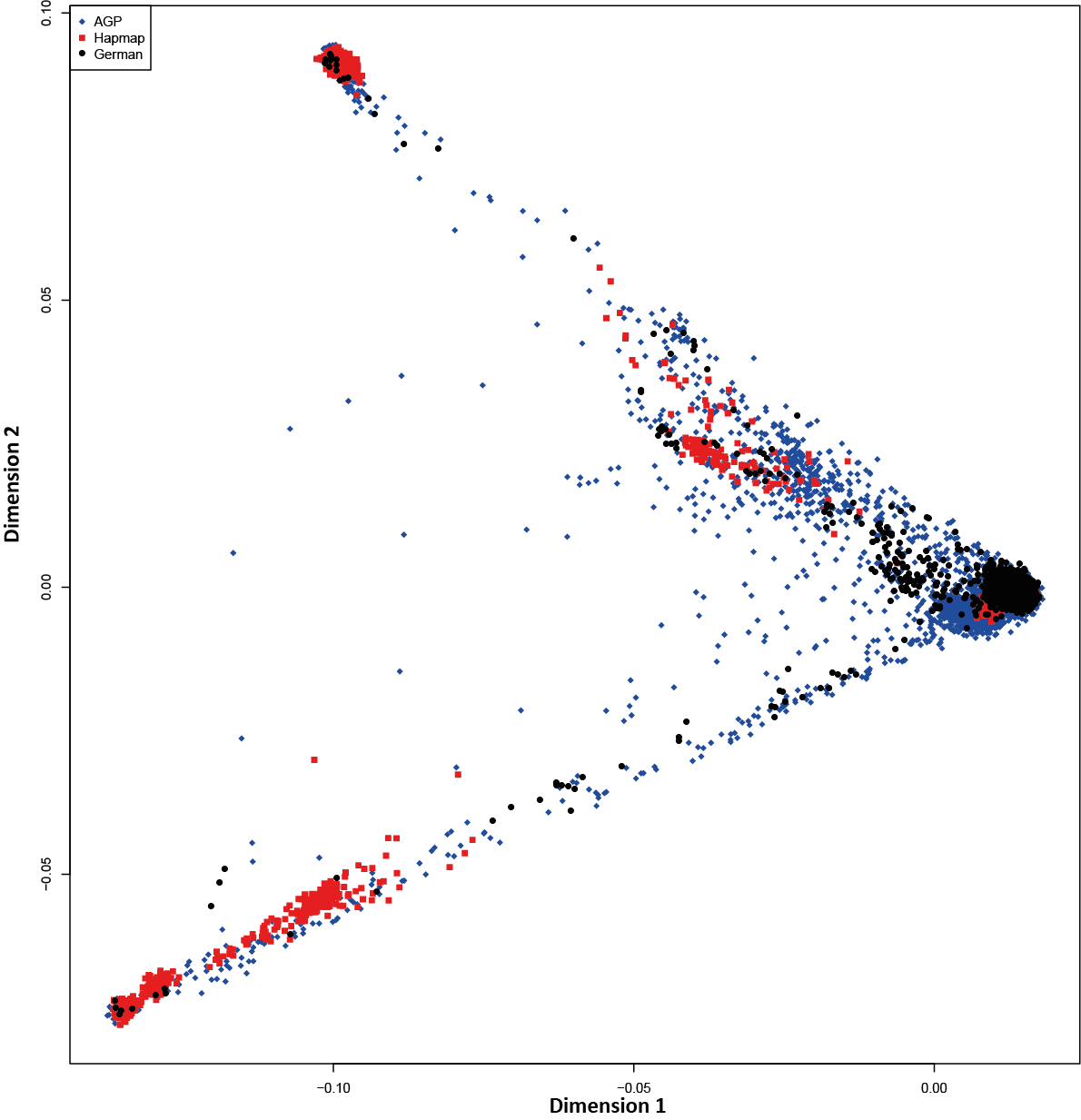


**Multidimensional scaling (MDS) plot:** The plot shows the top two dimensions of the multidimensional scaling performed for AGP, German and Hapmap cohorts.

## **Supplementary Figure 3**


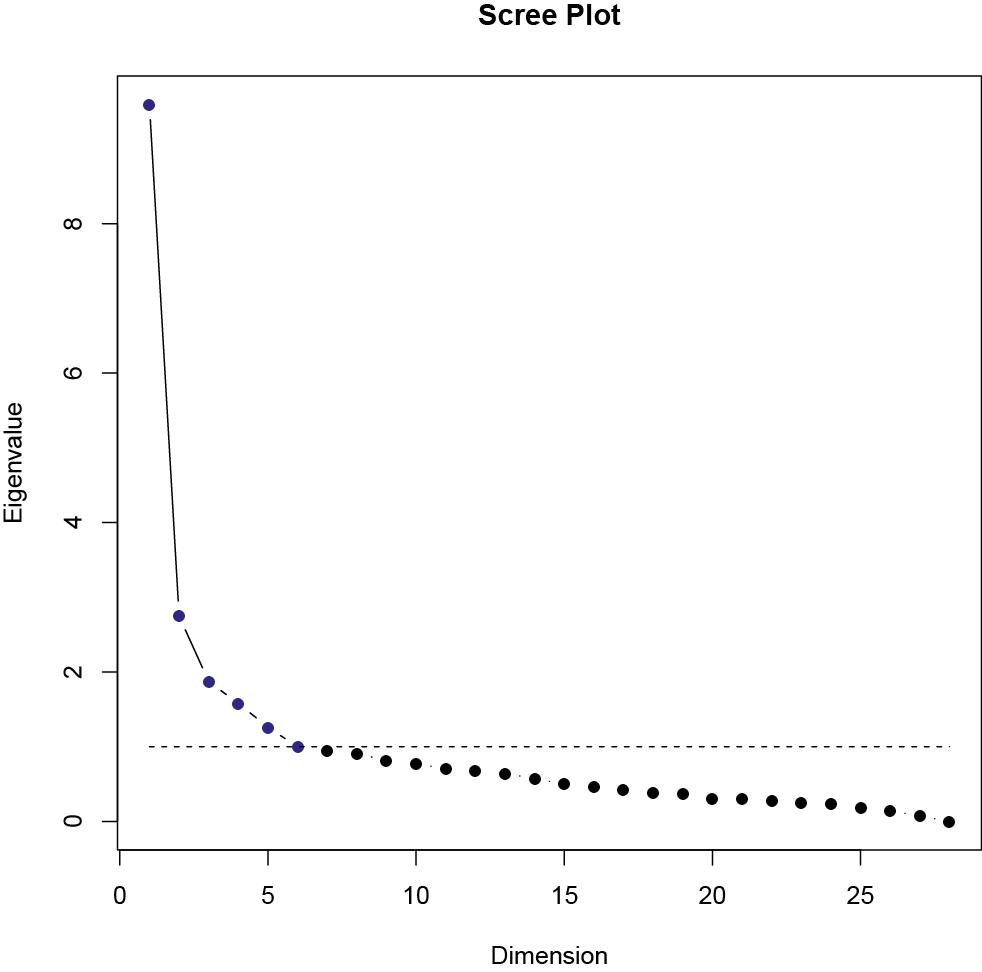


**Scree plot for exploratory factors analysis:** The number of factors (dimensions) with the corresponding eigenvalue at the y-axis and the number of factors at x-axis. In total six factors show an Eigenvalue larger than 1

## **Supplementary Figure 4**


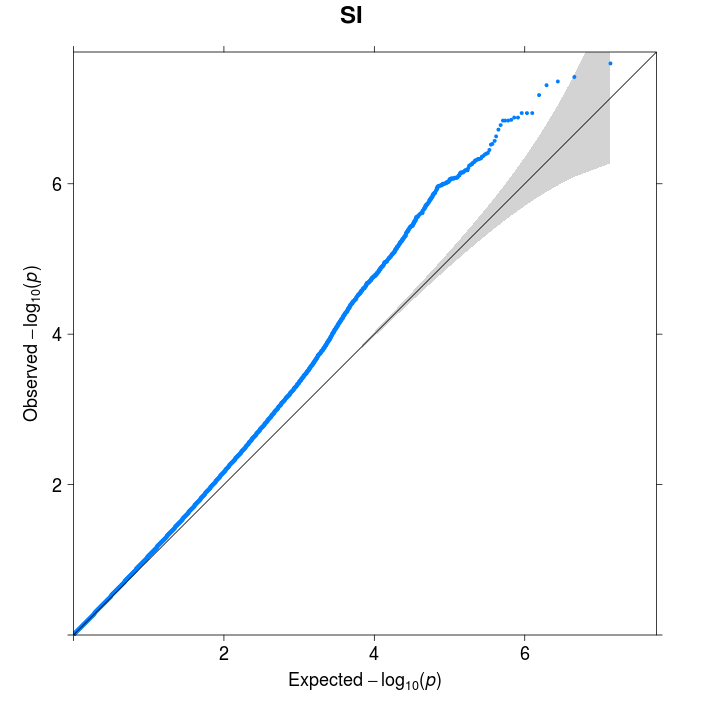

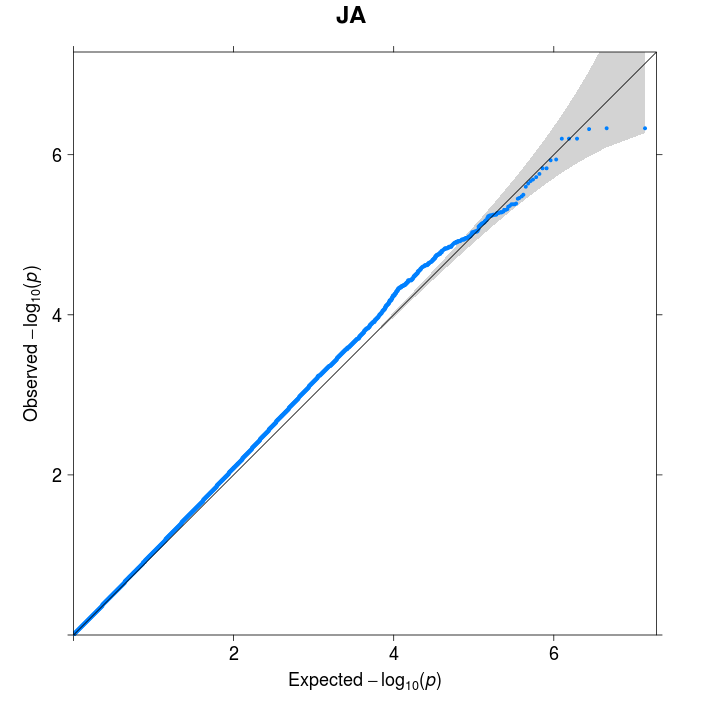

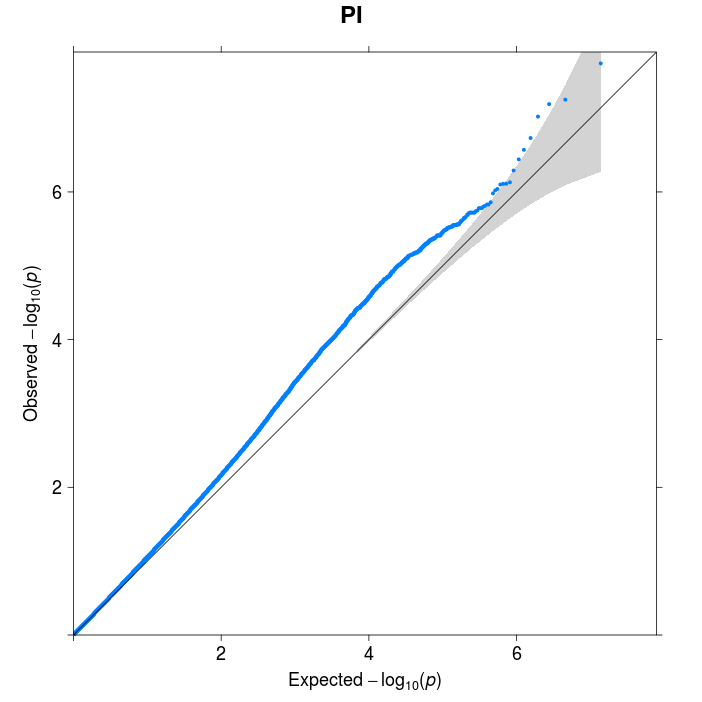

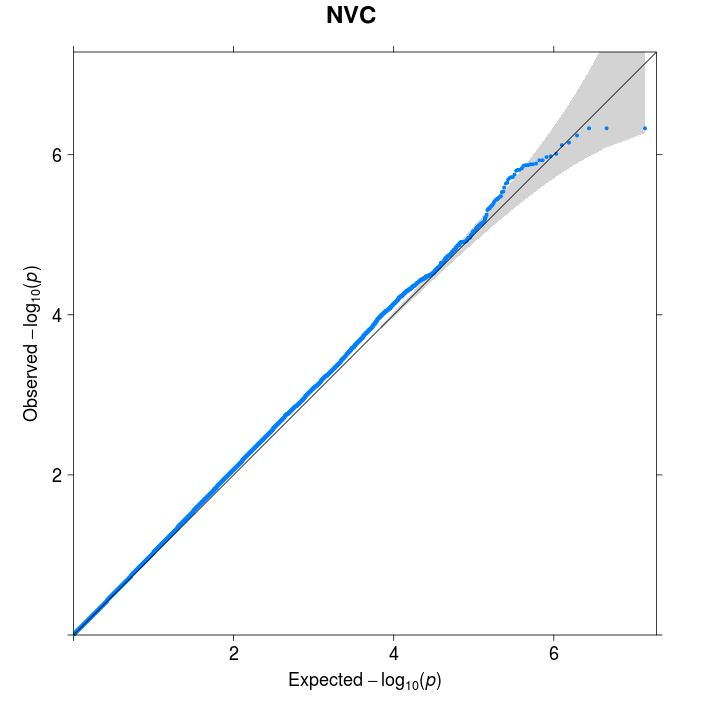

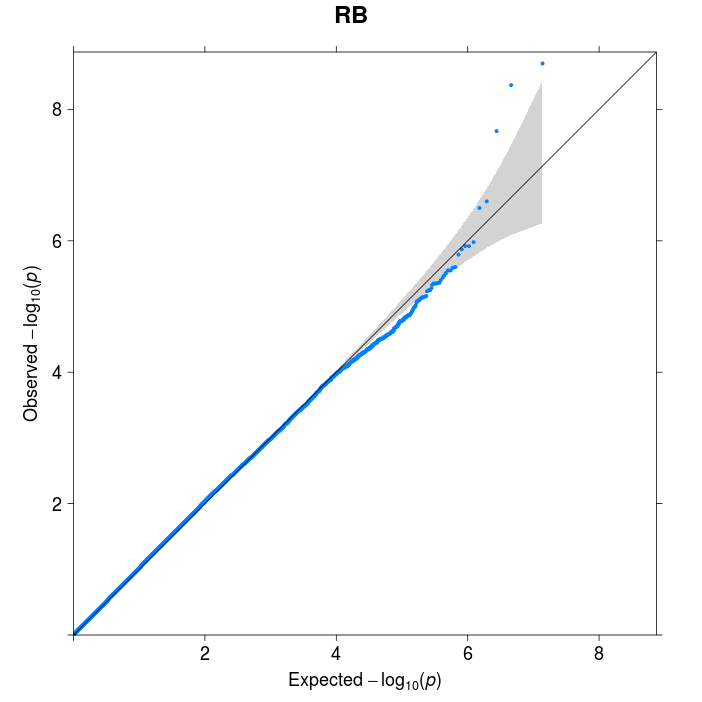

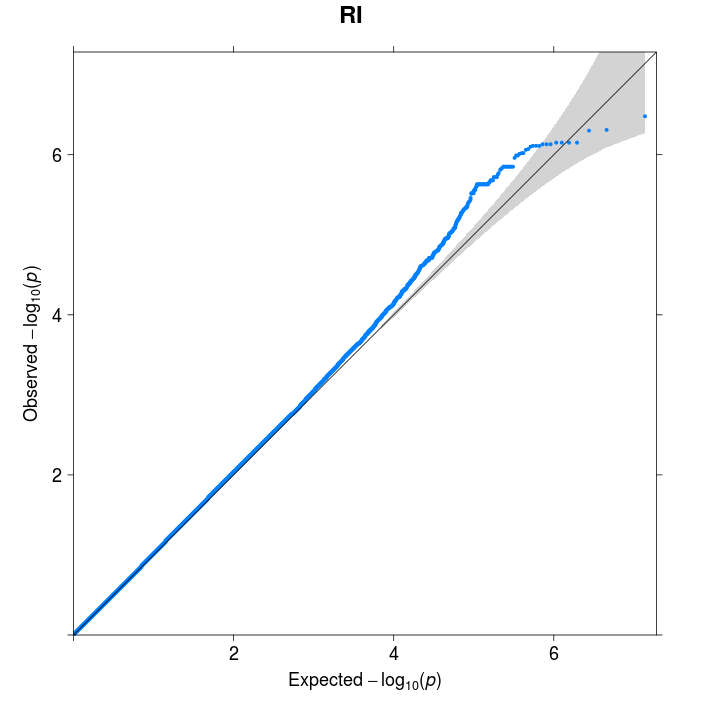


**QQplot for GWAS of combined cohort:** QQplot comparing the distribution between expected and observed –log_10_ association P-values.

## **Supplementary Figure 5**


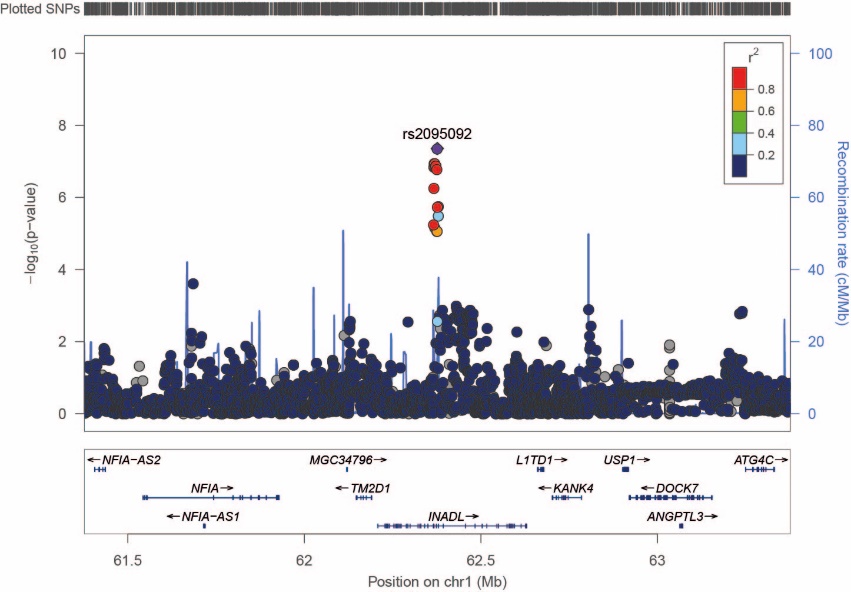

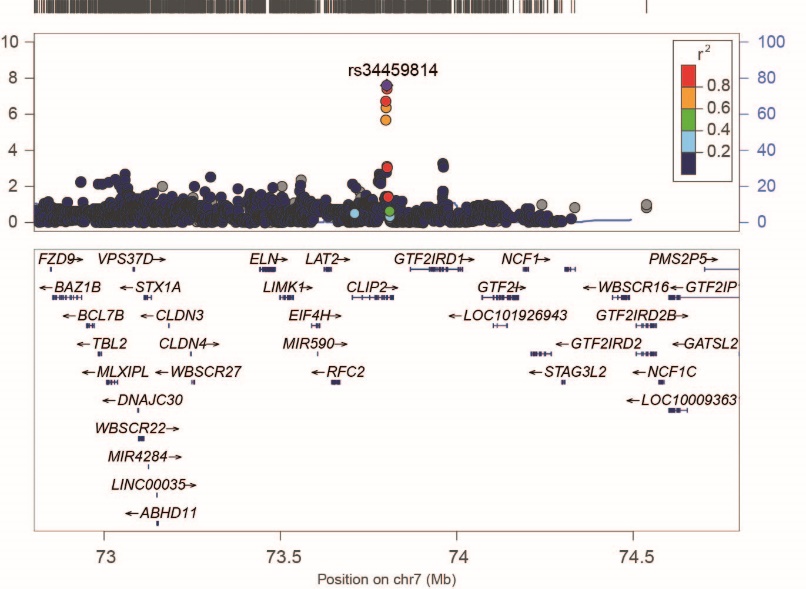

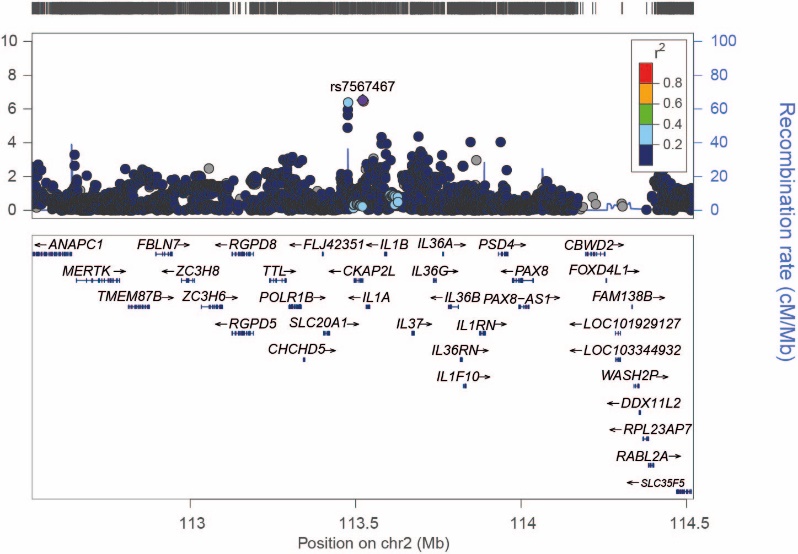


**SI_Peak 3**

**SI_Peak 1**

**SI_Peak 2**


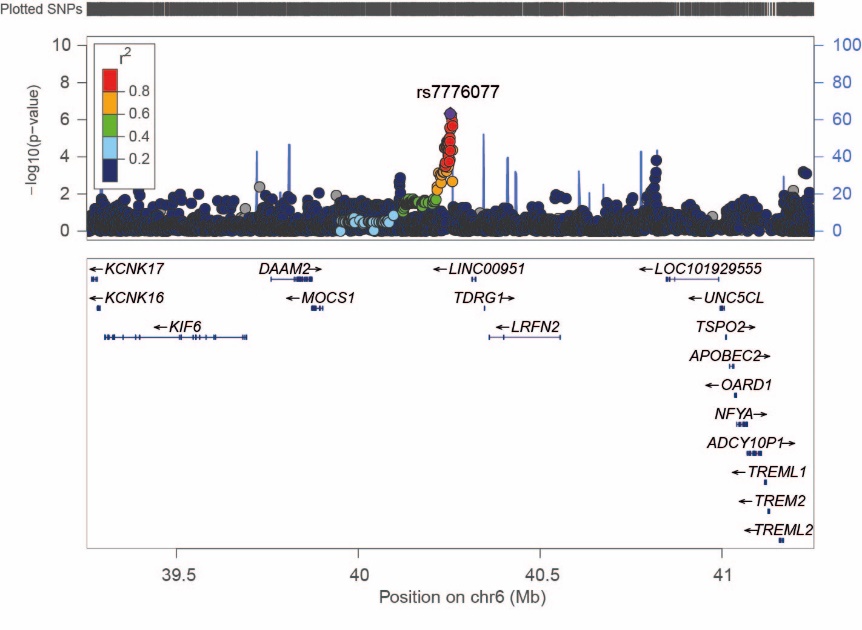

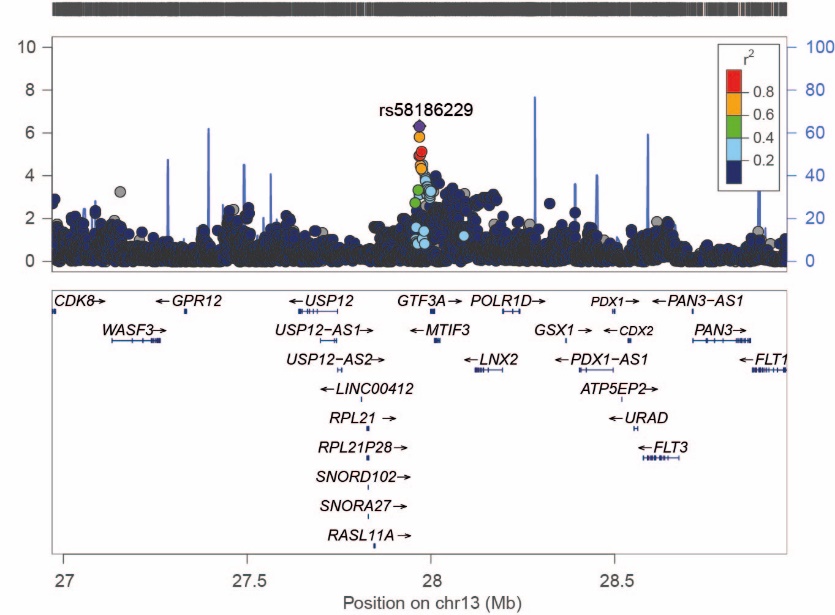

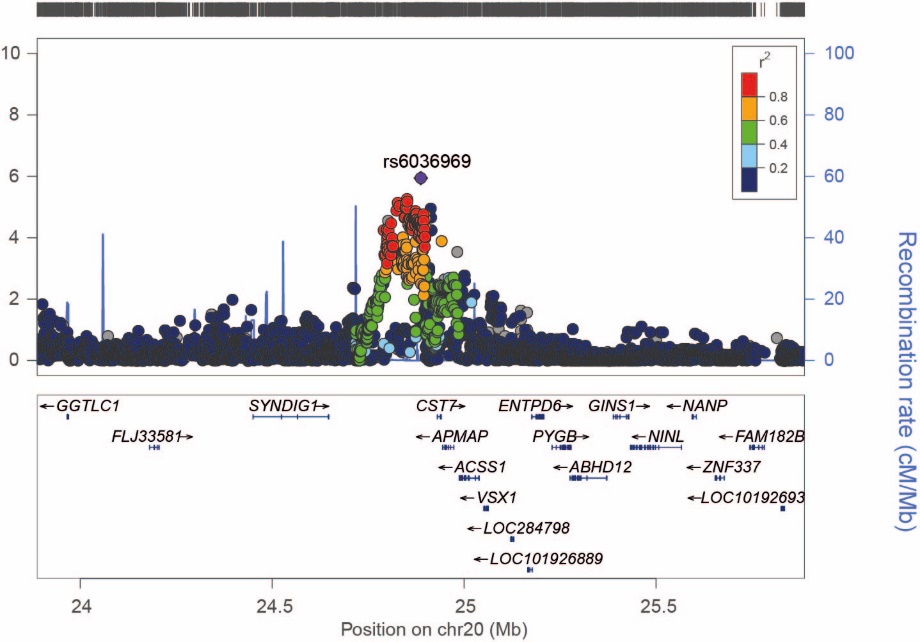


**JA_Peak 3**

**JA_Peak 1**

**JA_Peak 2**


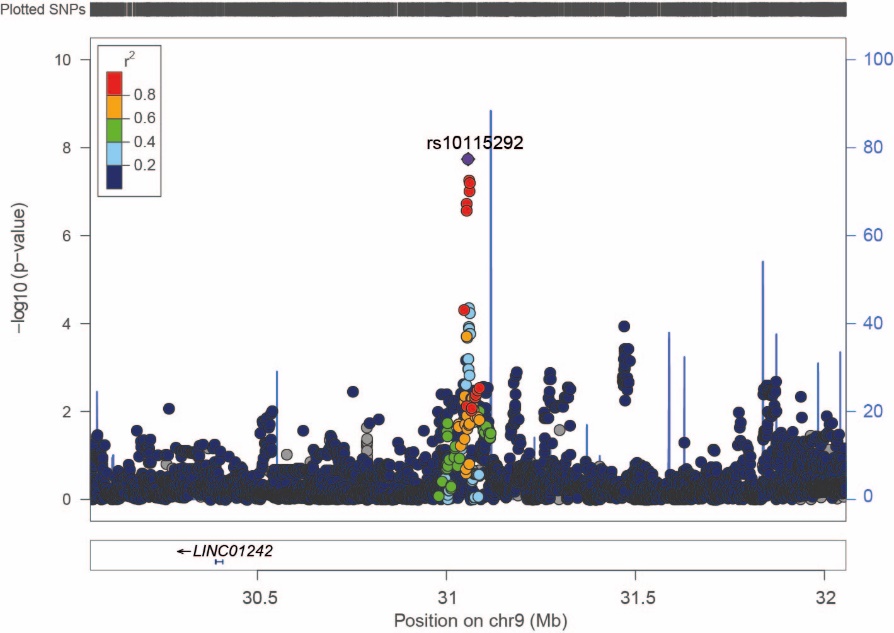

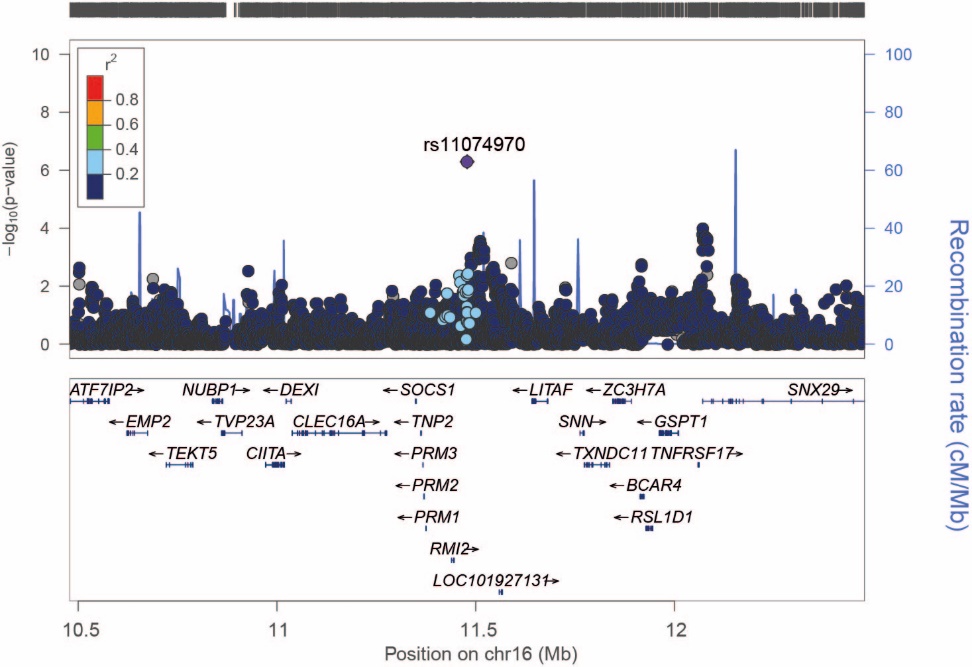

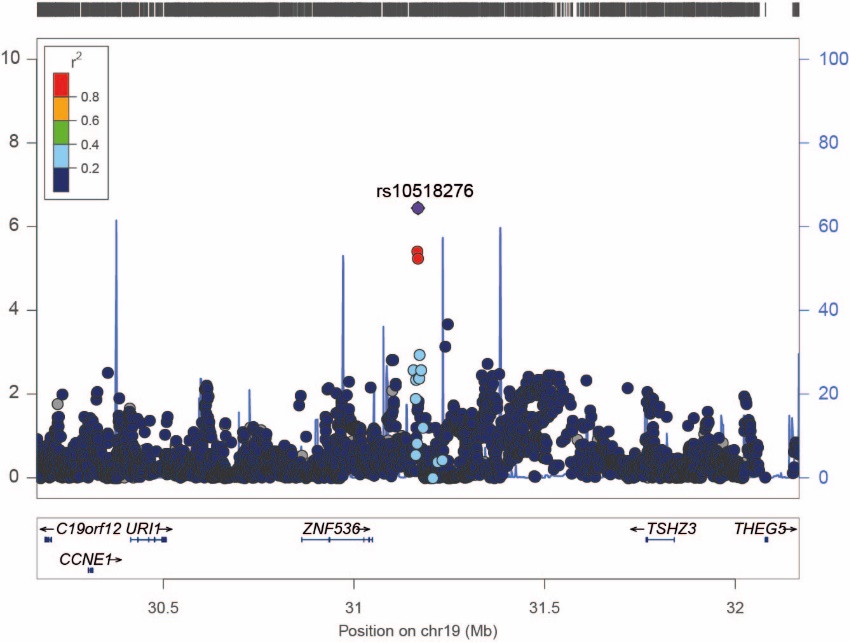


**PI_Peak 1**

**PI_Peak 2**

**PI_Peak 3**


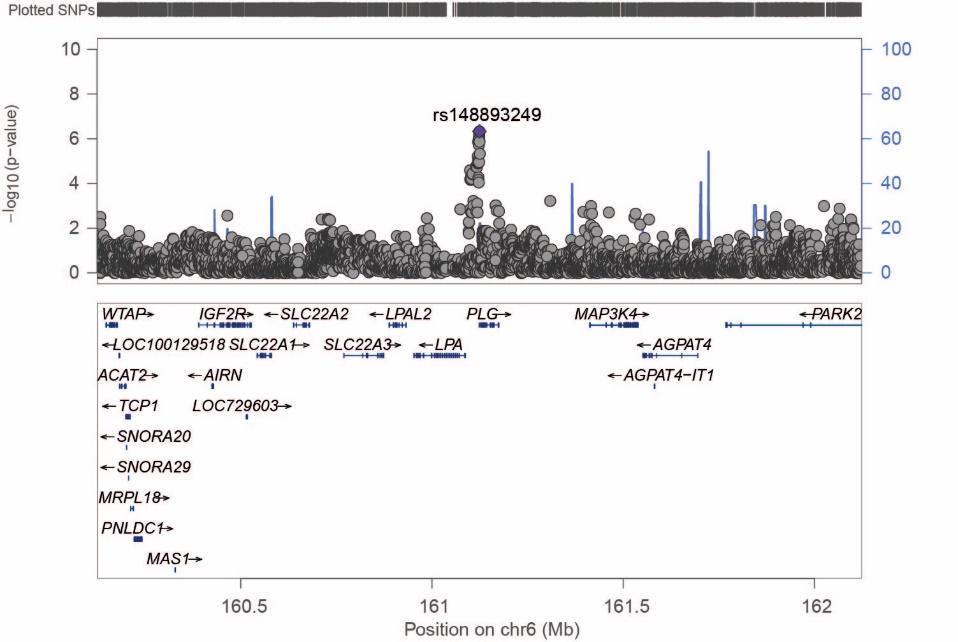

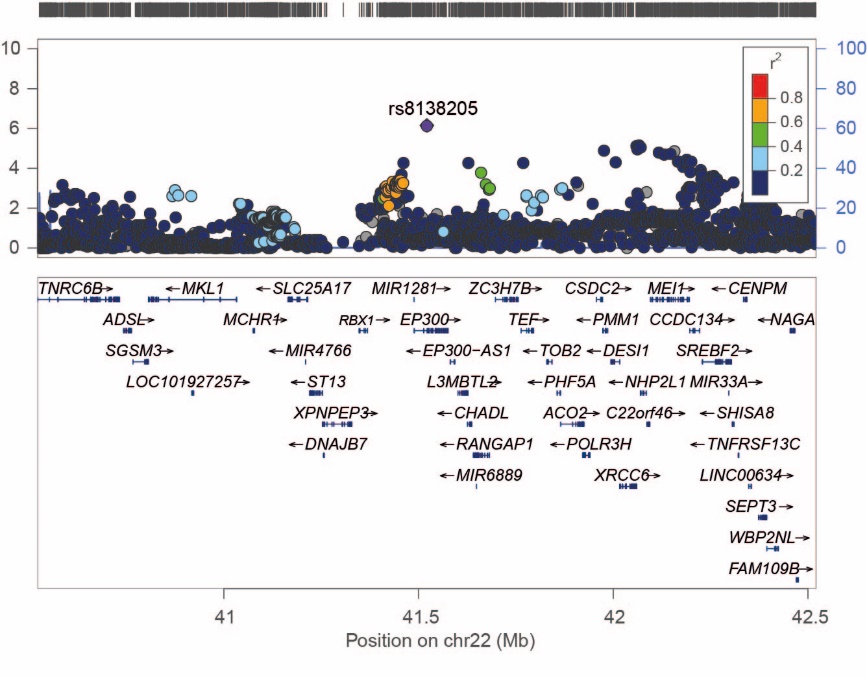

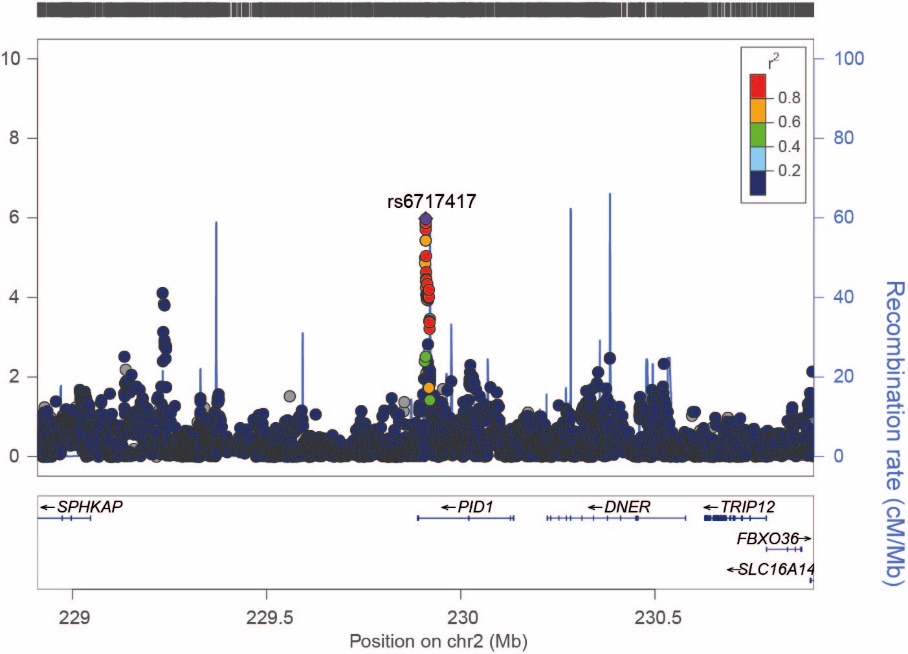


**NVC_Peak 1**

**NVC_Peak 2**

**NVC_Peak 3**


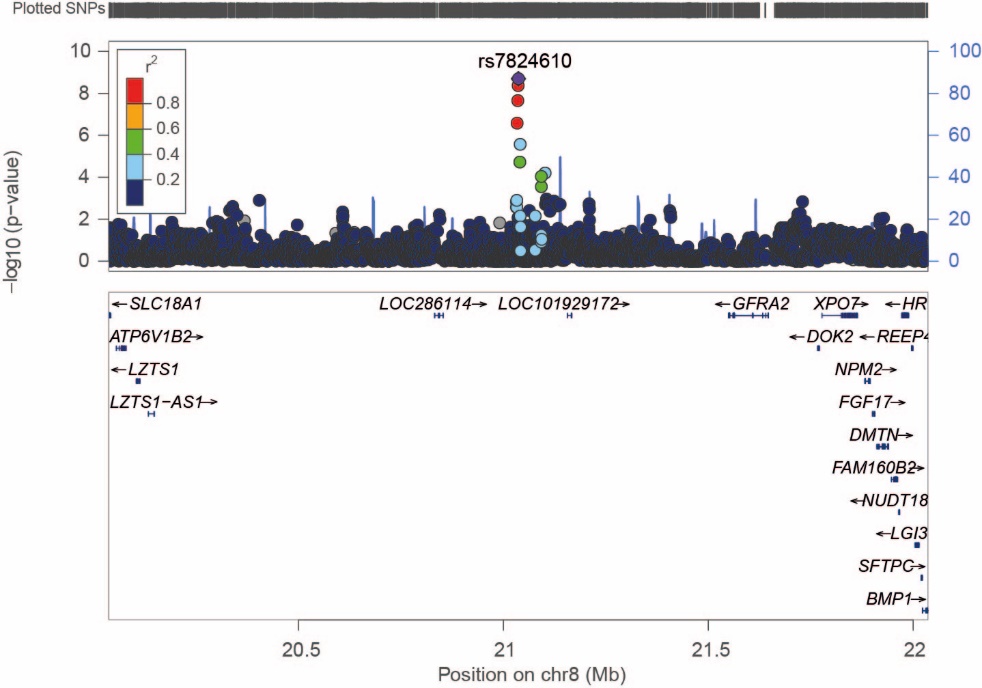

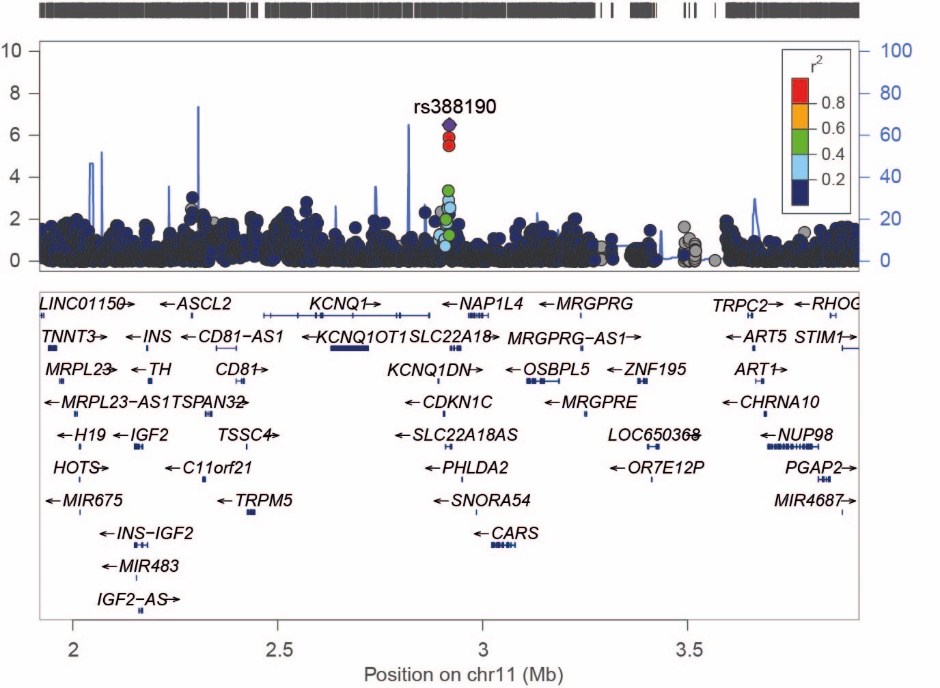

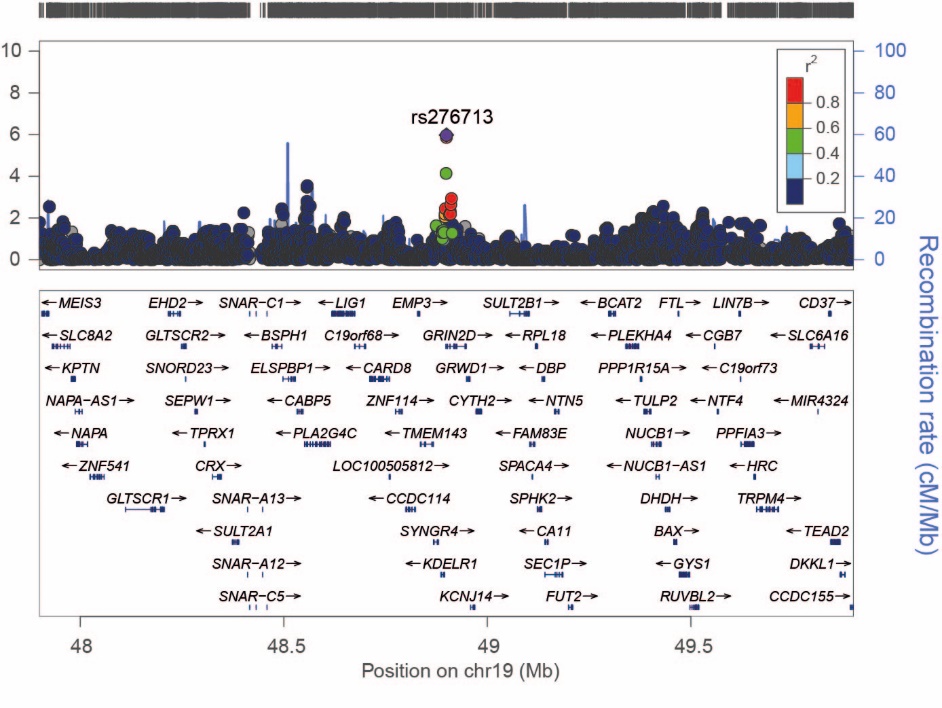


**RB_Peak 1**

**RB_Peak 2**

**RB_Peak 3**


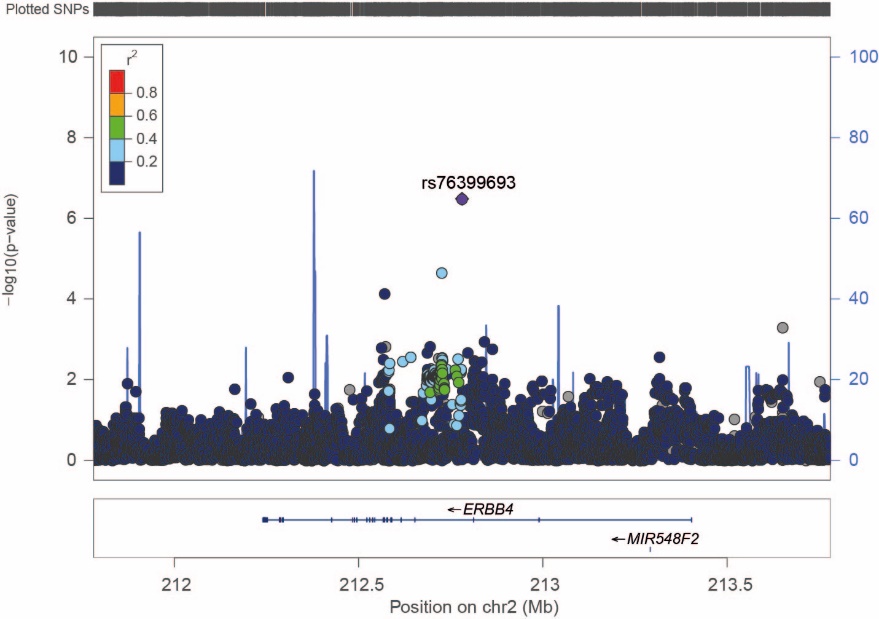

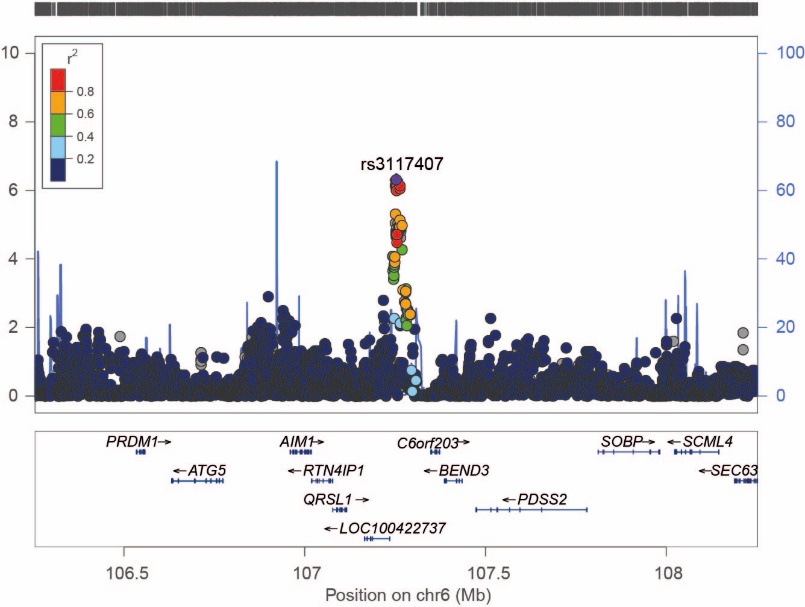

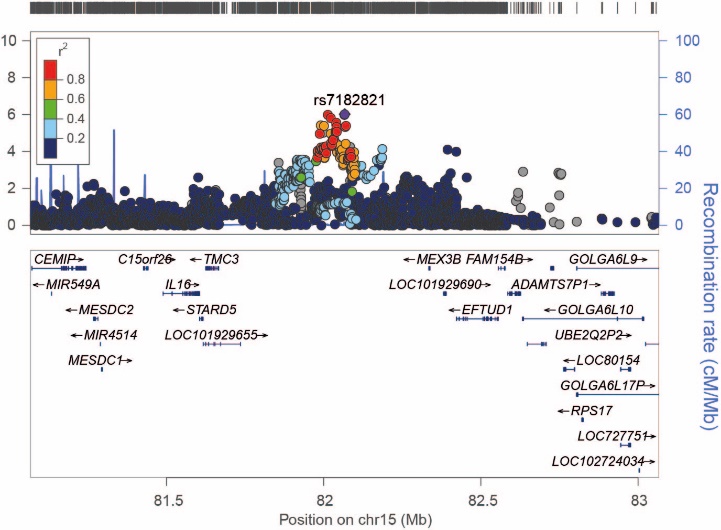


**RI_Peak 1**

**RI_Peak 2**

**RI_Peak 3**

**Locus plots of top three peaks for each subdomain** SI: Social Interaction; JA: Joint Attention; PI: Peer Interaction; NVC: Non-verbal Communication; RB: Repetitive sensory-motor Behavior; RI: Restricted Interest. The plot shows the genes in the region with there locations shown at the bottom, the SNP positions are shown at the top and the regional associations from GWAS are shown in the middle. The right axis gives the recombination rate shown as light blue line. The top associated SNPs are represented in purple color. The −log10 P values are shown for SNPs distributed in a 0.8-Mb genomic region that is centered where the most strongly associated signal is found, here shown as a purple diamond.

## **
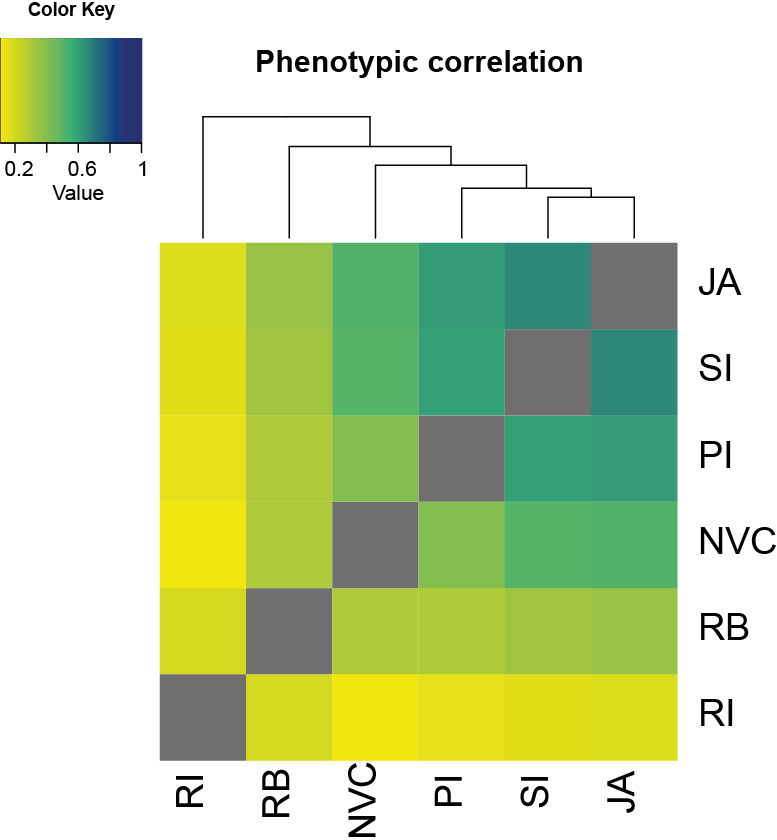
Supplementary Figure 6**

Phenotype correlation: The color of squares represents the intensity of correlations depicted with a color range of yellow to blue showing a range of lowest to highest correlation respectively. All correlations are significant P-value< 0.01.

## **
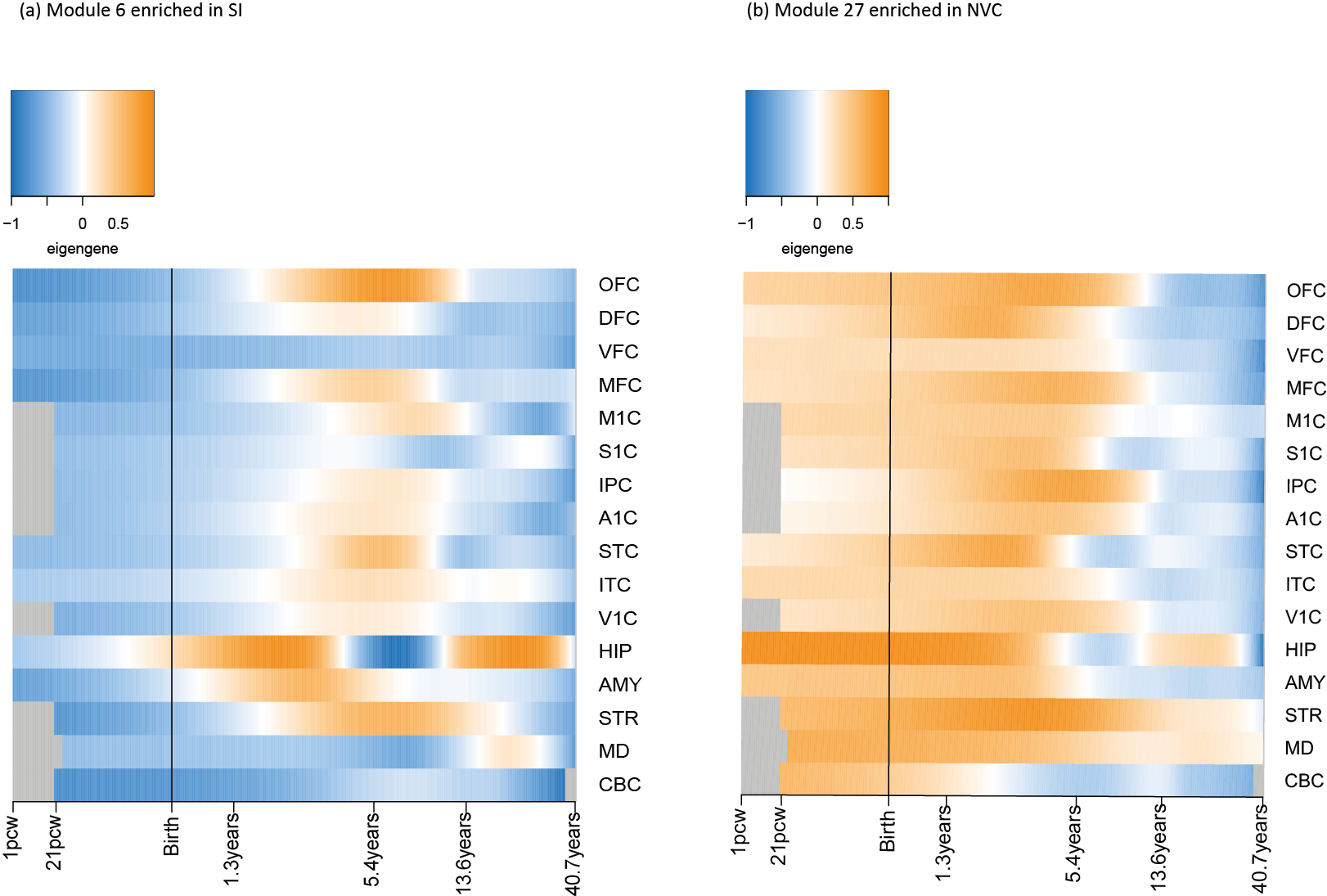
Supplementary Figure 7**

**Expression profiles of associated brain gene modules:** Eigengene expression of **(a) Module 6** enriched for genes implicated in SI (Social Interaction) **(b) Module 27** enriched for genes implicated in NVC (Non-verbal communication) where x-axis shows the developmental time frame and y-axis shows the different brain regions i.e. OFC: Orbital prefrontal cortex; DFC: Dorsolateral prefrontal cortex; VFC: Ventrolateral prefrontal cortex; MFC: Medial prefrontal cortex; M1C: Primary motor (M1) cortex; S1C: Primary somatosensory (S1) cortex; IPC: Posterior inferior parietal cortex; A1C: Primary auditory (A1) cortex, STC: Superior temporal cortex; ITC: Inferior temporal cortex; V1C: Primary visual (V1) cortex; HIP: Hippocampus; AMY: Amygdala; STR:Striatum; MD: Mediodorsal nucleus of the thalamus; CBC: Cerebellar cortex.

References

1. Freitag C.M., Staal W., Klauck S.M., Duketis E. & Waltes R. Genetics of autistic disorders: Review and clinical implications. *European child & adolescent psychiatry* **19**, 169–178 (2010).

2. Berument S.K., Rutter M., Lord C., Pickles A. & Bailey A. Autism screening questionnaire: diagnostic validity. *Br J Psychiatry* **175**, 444–451 (1999).

3. Lord C., Rutter M. & Le Couteur A. Autism Diagnostic Interview-Revised: A revised version of a diagnostic interview for caregivers of individuals with possible pervasive developmental disorders. *Journal of Autism and Developmental Disorders* **24**, 659–685 (1994).

4. Poustka F., Lisch S., Rühl D., Sacher A., Schmötzer G. & Werner K. The standardized diagnosis of autism, Autism Diagnostic Interview-Revised: Interrater reliability of the German form of the interview. *Psychopathology* **29**, 145–153 (1996).

5. Bölte S. & Poustka F. Diagnostische Beobachtungsskala für Autistische Störungen (ADOS): Erste Ergebnisse zur Zuverlässigkeit und Gültigkeit. *Z Kinder Jugendpsychiatr Psychother* **32**, 45–50 (2004).

6. Lord C. *et al.* The autism diagnostic observation schedule-generic: A standard measure of social and communication deficits associated with the spectrum of autism. *Journal of Autism and Developmental Disorders* **30**, 205–223 (2000).

7. Waltes R. *et al.* Common variants in genes of the postsynaptic FMRP signalling pathway are risk factors for autism spectrum disorders. *Human genetics* **133**, 781–792 (2014).

8. Waltes R. *et al.* Common EIF4E variants modulate risk for autism spectrum disorders in the high-functioning range. *Journal of Neural Transmission* **121**, 1107–1116 (2014).

9. Anney R. *et al.* A genome-wide scan for common alleles affecting risk for autism. *Human Molecular Genetics* **19**, 4072–4082 (2010).

10. Ripke S, Thomas B: Ricopili, 2011. Available at https://data.broadinstitute.org/mpg/ricopili/, accessed 08 Mar 2018.

11. Yousaf A. *et al.* Mapping the genetics of neuropsychological traits to the molecular network of the human brain using a data integrative approach **5** (2018).

12. Purcell S. *et al.* PLINK: A tool set for whole-genome association and population-based linkage analyses. *American Journal of Human Genetics* **81**, 559–575 (2007).

13. Rubeis S. de *et al.* Synaptic, transcriptional and chromatin genes disrupted in autism. *Nature* **515**, 209–215 (2014).

14. Auton A. *et al.* A global reference for human genetic variation. *Nature* **526**, 68–74 (2015).

15. Tyner C. *et al.* The UCSC Genome Browser database: 2017 update. *Nucleic Acids Research* **45**, D626-D634 (2017).

16. Delaneau O., Marchini J. & Zagury J.-F. A linear complexity phasing method for thousands of genomes. *Nature Methods* **9**, 179–181 (2011).

17. Das S. *et al.* Next-generation genotype imputation service and methods. *Nature Genetics* **48**, 1284–1287 (2016).

18. Sung Y.J., Wang L., Rankinen T., Bouchard C. & Rao D.C. Performance of genotype imputations using data from the 1000 Genomes Project. *Human Heredity* **73**, 18–25 (2012).

19. Visscher P.M. *et al.* Statistical power to detect genetic (co)variance of complex traits using SNP data in unrelated samples. *PLoS Genetics* **10**, e1004269 (2014).

20. Liu X.-Q. *et al.* Identification of genetic loci underlying the phenotypic constructs of autism spectrum disorders. *J Am Acad Child Adolesc Psychiatry* **50**, 687-696.e13 (2011).

21. Stef van Buuren & Karin Groothuis-Oudshoorn. mice: Multivariate Imputation by Chained Equations in R (2011).

22. Bodner T.E. What Improves with Increased Missing Data Imputations? *Structural Equation Modeling: A Multidisciplinary Journal* **15**, 651–675 (2008).

23. White I.R., Royston P. & Wood A.M. Multiple imputation using chained equations: Issues and guidance for practice. *Stat Med* **30**, 377–399 (2011).

24. William Revelle: psych: Procedures for Psychological, Psychometric, and Personality Research, 2017.

25. Tao Y., Gao H., Ackerman B., Guo W., Saffen D. & Shugart Y.Y. Evidence for contribution of common genetic variants within chromosome 8p21.2-8p21.1 to restricted and repetitive behaviors in autism spectrum disorders. *BMC Genomics* **17**, 163 (2016).

26. Bölte S. & Poustka F. Die Faktorenstruktur des Autismus Diagnostischen Interviews-Revision (ADI-R): Eine Untersuchung zur dimensionalen versus kategorialen Klassifikation autistischer Störungen. *Z Kinder Jugendpsychiatr Psychother* **29**, 221–229 (2001).

27. Kaiser H.F. The Application of Electronic Computers to Factor Analysis. *Educational and Psychological Measurement* **20**, 141–151 (1960).

28. Thorndike R.L. Who belongs in the family? *Psychometrika* **18**, 267–276 (1953).

29. Anderson T.W. & Rubin H. Statistical Inference in Factor Analysis. *Neyman, J., Ed., Proceedings of the Third Berkeley Symposium on Mathematical Statistics and Probability* **5**, 111–150 (1956).

30. Comrey A.L. Factor-analytic methods of scale development in personality and clinical psychology. *Journal of Consulting and Clinical Psychology* **56**, 754–761 (1988).

31. Guadagnoli E. & Velicer W.F. Relation of sample size to the stability of component patterns. *Psychol Bull* **103**, 265–275 (1988).

32. Tabachnick B.G. & Fidell L.S. *Using multivariate statistics* (Pearson Education: Boston, 2013).

33. Weissbrod O., Flint J. & Rosset S. Estimating SNP-Based Heritability and Genetic Correlation in Case-Control Studies Directly and with Summary Statistics. *American Journal of Human Genetics* **103**, 89–99 (2018).

34. Yang J., Lee S.H., Goddard M.E. & Visscher P.M. GCTA: A tool for genome-wide complex trait analysis. *American Journal of Human Genetics* **88**, 76–82 (2011).

35. Bulik-Sullivan B.K. *et al.* LD Score regression distinguishes confounding from polygenicity in genome-wide association studies. *Nature Genetics* **47**, 291–295 (2015).

36. Yang J. *et al.* Genome partitioning of genetic variation for complex traits using common SNPs. *Nature Genetics* **43**, 519–525 (2011).

37. Lee S.H., Yang J., Goddard M.E., Visscher P.M. & Wray N.R. Estimation of pleiotropy between complex diseases using single-nucleotide polymorphism-derived genomic relationships and restricted maximum likelihood. *Bioinformatics* **28**, 2540–2542 (2012).

38. Euesden J., Lewis C.M. & O'Reilly P.F. PRSice: Polygenic Risk Score software. *Bioinformatics* **31**, 1466–1468 (2015).

39. Leeuw C.A. de, Mooij J.M., Heskes T. & Posthuma D. MAGMA: Generalized gene-set analysis of GWAS data. *PLoS Comput Biol* **11**, e1004219 (2015).

40. Zambon A.C. *et al.* GO-Elite: A flexible solution for pathway and ontology over-representation. *Bioinformatics* **28**, 2209–2210 (2012).

41. Kang H.J. *et al.* Spatio-temporal transcriptome of the human brain. *Nature* **478**, 483–489 (2011).

42. Bahl E., Koomar T. & Michaelson J.J. cerebroViz: An R package for anatomical visualization of spatiotemporal brain data. *Bioinformatics* **33**, 762–763 (2017).

43. Csardi G. & Nepusz T. The igraph software package for complex network research (2006).

44. Hu L.‐t. & Bentler P.M. Cutoff criteria for fit indexes in covariance structure analysis: Conventional criteria versus new alternatives. *Structural Equation Modeling: A Multidisciplinary Journal* **6**, 1–55 (1999).

45. MacCallum R.C., Browne M.W. & Sugawara H.M. Power analysis and determination of sample size for covariance structure modeling. *Psychol Methods* **1**, 130–149 (1996).
